# Beta regression model nonlinear in the parameters with additive measurement errors in variables

**DOI:** 10.1371/journal.pone.0254103

**Published:** 2021-07-29

**Authors:** Daniele de Brito Trindade, Patrícia Leone Espinheira, Klaus Leite Pinto Vasconcellos, Jalmar Manuel Farfán Carrasco, Maria do Carmo Soares de Lima

**Affiliations:** 1 Instituto Federal de Educação Ciência e Tecnologia Baiano Guanambi, Guanamb, BA, Brazil; 2 Departamento de Estatística, Universidade Federal de Pernambuco, Recife, Pernambuco, Brazil; 3 Departamento de Estatística, Universidade Federal da Bahia, Salvador, Bahia, Brazil; Universita degli Studi di Genova, ITALY

## Abstract

We propose in this paper a general class of nonlinear beta regression models with measurement errors. The motivation for proposing this model arose from a real problem we shall discuss here. The application concerns a usual oil refinery process where the main covariate is the concentration of a typically measured in error reagent and the response is a catalyst’s percentage of crystallinity involved in the process. Such data have been modeled by nonlinear beta and simplex regression models. Here we propose a nonlinear beta model with the possibility of the chemical reagent concentration being measured with error. The model parameters are estimated by different methods. We perform Monte Carlo simulations aiming to evaluate the performance of point and interval estimators of the model parameters. Both results of simulations and the application favors the method of estimation by maximum pseudo-likelihood approximation.

## 1 Introduction

Regression models for dependent variables that assume values in the unit interval have been proving quite important in the literature, with a special highlight to the beta regression model proposed by [1] and generalized by [2] whom proposed the nonlinear beta regression model. In the above mentioned models traditionally all covariates are considered fixed, not random, without measurement error. In practice, covariates may not be observed directly or may be subject to measurement errors. It is important to emphasize that if this assumption is not respected, unreliable inferential results shall be obtained [3]. In these circumstances, regression models with measurement errors are usually defined and structured so that the mean response is explained by covariates *x*_*t*_ whose measurements are inaccurate. Thus, instead of the true value of *x*_*t*_, the value of another predictor variable, *w*_*t*_, which is associated with a measurement error, is considered.

It is reasonable that in regression models for dependent variables assuming values in the unit interval, some of the covariates are not observed directly, but acquired with possible measurement errors. Thus, [4] proposed the beta regression model with measurement errors, in which covariates measured with errors can enter in the mean and precision submodels described by linear predictors. A possible extension to the model proposed by [4] is to consider a nonlinear modeling for the parameters. In fact, a real problem has shown the necessity of this modeling proposal. The interest of the problem lies in modeling the percentage of crystallinity of a chemical reagent based on different concentrations of vanadium and steam, considering two values for temperature. It is expected that the higher the concentrations of vanadium and steam, the lower the percentage of crystallinity. The loss of crystallinity is a negative effect of the process and precise knowledge of how vanadium concentration destroys this crystallinity is one of the most chief goals of this regression modeling. As we shall describe in the application, the measurement of vanadium concentration may be inaccurate, which characterizes the possibility of this covariate to present measurement error. These data were modeled by [5] based on beta and simplex nonlinear regressions in which the vanadium was taken as a fixed covariate. In our application the nonlinearity is related with steam, that is here treated as a fixed covariate.

Nonlinear models with measurement errors have been being recently studied, as shown in the literature; see [6–9]. Our aim here is propose a particular class of nonlinear beta regression with measured errors, in which the nonlinearity is on a parameter related to a fixed covariate. We consider three methods for the estimation of the parameters, namely: approximate maximum likelihood, approximate pseudo maximum likelihood and regression calibration. We compare these three methods with the estimation of the naive model, which considers that the regression does not have covariates measured in error, that is, it considers the classical nonlinear beta regression model. We evaluate the properties of point and interval estimators for the four estimation methods considered, based on Monte Carlo simulations and different scenarios. Both the simulations and our application pointed out that the estimation method by approximate pseudo maximum likelihood is the one that presents the best performance. Furthermore, recently [10] proposed the simplex regression models with measurement error, and as a future research we shall extend this proposal to nonlinear case.

## 2 Model and estimation methods

Consider *n* independent random variables *y*_1_, …, *y*_*n*_ such that each *y*_*t*_, *t* = 1, …, *n* is beta distributed with density

$$\begin{array}{c} f\left(y_{t};\mu_{t},\phi_{t}\right)=\Gamma (\phi_{t}){\left(\Gamma \left(\mu_{t}\phi_{t}\right)\Gamma \left(1-\mu_{t}\right)\phi_{t}\right)}^{-1}y_{t}^{\mu_{t}\phi_{t}-1}{\left(1-y_{t}\right)}^{\left(1-\mu_{t}\right)\phi_{t}-1},\;0<y_{t}<1, \end{array}$$ (1)

0 < *μ*_*t*_ < 1, *ϕ*_*t*_ > 0, E(*y*_*t*_) = *μ*_*t*_ and Var(*y*_*t*_) = *μ*_*t*_(1 − *μ*_*t*_)/1 + *ϕ*_*t*_. The class of nonlinear beta regression models here with measurement errors here proposed considers

$$\begin{array}{c} g_{1}\left(\mu_{t}\right)=\eta_{1t}=f_{1}\left(z_{t}^{⊤},x_{t}^{⊤};\alpha ,\beta \right),\;g_{2}\left(\phi_{t}\right)=\eta_{2t}=f_{2}\left(v_{t}^{⊤},m_{t}^{⊤};\gamma ,\lambda \right), \end{array}$$ (2)

where *α* = (*α*_1_, …, *α*_*p*_)^⊤^ ∈ IR^*p*^, *β* = (*β*_1_, …, *β*_*r*_)^⊤^ ∈ IR^*r*^, $\gamma =\left(\gamma_{1},\ldots ,\gamma_{\overset{ˇ}{q}}\right)\in I\;R^{\overset{ˇ}{q}}$, λ = (λ_1_, …, λ_*s*_)^⊤^ ∈ IR^*s*^ are unknown parameters, $\left(p+r\right)+\left(\overset{ˇ}{q}+s\right)<n$, $z_{t}^{⊤}=\left(z_{t1},\ldots ,z_{tp}\right)$ and $v_{t}^{⊤}=\left(\upsilon_{t1},\ldots ,\upsilon_{t\overset{ˇ}{q}}\right)$ are fixed and unknown vectors of observations and $x_{t}^{⊤}=\left(x_{t1},\ldots ,x_{tr}\right)$, $m_{t}^{⊤}=\left(m_{t1},\ldots ,m_{ts}\right)$ are vectors of covariates not directly observed or associated to measurement errors. The link functions *g*_1_(⋅):(0, 1)→IR and *g*_2_(⋅):(0, ∞)→IR are strictly monotonic, continuous and twice differentiable. Further, *f*_1_(⋅) and *f*_2_(⋅) are differentiable functions with Jacobian matrices **F**_1_ = ∂*η*_1_/∂*α*, **F**_2_ = ∂*η*_1_/∂*β*, **F**_3_ = ∂*η*_2_/∂*γ* and **F**_4_ = ∂*η*_2_/∂λ having ranks *p*, *r*, $\overset{ˇ}{q}$ and *s*, respectively. We should point out that the method proposed here is not applicable to models with random effects. Finally, the parameter defining the degree of variability of precision is here defined as $\delta =\max_{\left\{t=1,\ldots ,n\right\}}\phi_{t}/\min_{\left\{t=1,\ldots ,n\right\}}\phi_{t}$. For the model with constant precision, *ϕ*_1_ = … = *ϕ*_*n*_ = *ϕ*, and, therefore, *δ* = 1.

Following [4] we consider that the variables with measurement errors in the submodels for mean and precision are the same. Also, the vector of random measurements **x**_*t*_ is not directly observed. The observed vector is **w**_*t*_ = (*w*_*t*1_, …, *w*_*tr*_)^⊤^, which we here suppose related to **x**_*t*_ in the form **w**_*t*_ = *τ*_**1**_ + *τ*_**2**_∘**x**_*t*_ + **e**_*t*_, where **e**_*t*_ is a vector of random errors, *τ*_1_ = (*τ*_11_, …, *τ*_1*r*_)^⊤^ ∈ IR^*r*^ and *τ*_2_ = (*τ*_21_, …, *τ*_2*r*_)^⊤^ ∈ IR^*r*^ are vectors of unknown parameters and “∘” represents the Hadamard product; for two vectors **u**_1_ = (*u*_11_, …, *u*_1*r*_)^⊤^ and **u**_2_ = (*u*_21_, …, *u*_2*r*_)^⊤^ of same dimension, **u**_1_∘**u**_2_ = (*u*_11_ *u*_21_, …, *u*_1*r*_ *u*_2*r*_)^⊤^. We may assume *τ*_1_ to be a vector of zeros, as we may assume *τ*_2_ to be a vector of ones. However, we can also assume them to be unknown parameters to be estimated, as we shall see in the application.

Hence, our vector of unknown parameters is $\Psi ={\left(\theta^{⊤},\xi^{⊤},\sigma_{e}^{2⊤}\right)}^{⊤}$, where *θ* = (*α*^⊤^, *β*^⊤^, *γ*^⊤^, λ^⊤^)^⊤^ and ${\left(\xi^{⊤},\sigma_{e}^{2⊤}\right)}^{⊤}={\left(\mu^{⊤},\sigma_{x}^{2⊤},\sigma_{e}^{2⊤}\right)}^{⊤}$ are, respectively, the vectors of interest and nuisance parameters. The joint density function of (*y*_*t*_, **w**_*t*_) is

$$\begin{array}{cc} f(y_{t},w_{t};\Psi )= & \int_{-\infty }^{\infty }\cdots \int_{-\infty }^{\infty }f\left(y_{t}|x_{t};\theta \right)f\left(w_{t}|x_{t};\sigma_{e}^{2}\right)f\left(x_{t};\xi \right)dx_{t}. \end{array}$$ (3)

In (3), *f*(*y*_*t*_|**x**_*t*_;*θ*) represents a density for a beta distribution, $f(w_{t}|x_{t};\sigma_{e}^{2})$ is a conditional density of **w**_*t*_ given **x**_*t*_ and *f*(**x**_*t*_, *ξ*) is a density for the explanatory variable **x**_*t*_. Observe that, to obtain (3), we must assume that *y*_*t*_ is independent of **w**_*t*_ given **x**_*t*_, that is, the distribution of *y*_*t*_ given (**w**_*t*_, **x**_*t*_) involves only **x**_*t*_ [8]. Therefore, the log-likelihood function for the *n* independent observations is

$$\begin{array}{cc} \ell (\Psi ) & =\sum_{t=1}^{n}\log\int_{-\infty }^{\infty }\cdots \int_{-\infty }^{\infty }f\left(y_{t}|x_{t};\theta \right)f\left(x_{t}|w_{t};\xi ,\sigma_{e}^{2}\right)f\left(w_{t};\xi ,\sigma_{e}^{2}\right)dx_{t}\\ = & \sum_{t=1}^{n}\log f\left(w_{t};\xi ,\sigma_{e}^{2}\right)+\sum_{t=1}^{n}\log\int_{-\infty }^{\infty }\cdots \int_{-\infty }^{\infty }f\left(y_{t}|x_{t};\theta \right)f\left(x_{t}|w_{t};\xi ,\sigma_{e}^{2}\right)dx_{t}, \end{array}$$ (4)

The log-likelihood above involves integrals that are analytically unsolvable, which means that approximation methods need to be used. We will consider here three different approaches to estimate the parameters and, aiming to describe each one of the three methods, we will suppose, for illustration purposes, that only *x*_*t*_ is measured with error. However, it is important to stress that the methods here used can be easily generalized to *r* covariates with measurement errors.

From now on, we make the following assumptions:

$$y_{t}|x_{t}∼Beta\left(\mu_{t},\phi_{t}\right),\;w_{t}=x_{t}+e_{t},\;x_{t}\overset{\text{ind}}{∼}N\left(\mu_{x},\sigma_{x}^{2}\right),\;\;e_{t}\overset{\text{ind}}{∼}N\left(0,\sigma_{e}^{2}\right),$$

and, finally, *x*_*t*_ and *e*_*t*′_, with *t*, *t*′ = 1, …, *n*, are independent. This is a *structural model* for which the vector of non-observed variables follows a normal distribution. It is important here to stress that the models for *μ*_*t*_ and *ϕ*_*t*_, *t* = 1, …, *n* are based in (2). From the above assumptions, we have

$$\begin{array}{cc} & w_{t}\overset{\text{ind}}{∼}N\left(\mu_{x},\sigma_{x}^{2}+\sigma_{e}^{2}\right),\;x_{t}|w_{t}\overset{\text{ind}}{∼}N\left(\mu_{x_{t}|w_{t}},\sigma_{x_{t}|w_{t}}^{2}\right), \end{array}$$ (5)

$$\begin{array}{cc} & \mu_{x_{t}|w_{t}}=\mu_{x}+k_{x}\left(w_{t}-\mu_{x}\right),\;\sigma_{x_{t}|w_{t}}^{2}=\sigma_{e}^{2}k_{x},\;k_{x}=\sigma_{x}^{2}/\left(\sigma_{x}^{2}+\sigma_{e}^{2}\right), \end{array}$$ (6)

where *k*_*x*_ is known as the **reliability coefficient**. An additional assumption is that the variance $\sigma_{e}^{2}$ of the measurement error is known. This assumption is necessary to avoid identifiability problems. When we have replicas for *w*_*t*_, it is possible to use the sample variance of *w*_*t*_ to estimate the variance of the measurement error.

In order to establish the log-likelihood for the model to be studied, we use the results in (5) and also that $\sigma_{e}^{2}={\hat{\sigma }}_{e}^{2}$, from which we obtain

$$\begin{array}{cc} \ell (\Psi ) & =\sum_{t=1}^{n}\ell_{1t}\left(\xi ,{\hat{\sigma }}_{e}^{2}\right)+\sum_{t=1}^{n}\ell_{2t}\left(\theta ,\xi ,{\hat{\sigma }}_{e}^{2}\right),\\ \ell_{1t}\left(\xi ,{\hat{\sigma }}_{e}^{2}\right) & =\log f\left(w_{t};\xi ,{\hat{\sigma }}_{e}^{2}\right)=-\frac{1}{2}\log\left[2\pi \left(\sigma_{x}^{2}+{\hat{\sigma }}_{e}^{2}\right)\right]-\frac{1}{2(\sigma_{x}^{2}+{\hat{\sigma }}_{e}^{2})}{\left(w_{t}-\mu_{x}\right)}^{2}, \end{array}$$ (7)

$$\begin{array}{cc} \ell_{2t}\left(\theta ,\xi ,{\hat{\sigma }}_{e}^{2}\right) & =\log\int_{-\infty }^{\infty }f\left(y_{t}|x_{t};\theta ,\xi ,{\hat{\sigma }}_{e}^{2}\right)\frac{1}{\sqrt{2\pi \sigma_{x_{t}|w_{t}}^{2}}}\exp[-\frac{{\left(x_{t}-\mu_{x_{t}|w_{t}}\right)}^{2}}{2\sigma_{x_{t}|w_{t}}^{2}}]dx_{t}. \end{array}$$ (8)

We also suppose that the log-likelihood is a concave function of the parameters to be estimated. The estimators we shall propose have no closed form. Therefore, we shall use the nonlinear optimization method Fisher’s scoring with initial values proposed by [11] to the nonlinear beta regression models.

### 2.1 Estimation by approximate maximum likelihood

The integral in (8) can be approximated using the Gauss-Hermite quadrature, given by

$$\begin{array}{c} \int_{-\infty }^{\infty }e^{-x^{2}}f\left(x\right)dx\approx \sum_{q=1}^{Q}\nu_{q}f\left(s_{q}\right), \end{array}$$ (9)

where *s*_*q*_ and *ν*_*q*_ are, respectively, the *q*-th zero and weight of the *Q*-th order orthogonal Hermite polynomial, *Q* being the number of points for quadrature [12]. Using the change of variable $u_{t}=\left(x_{t}-\mu_{x_{t}|w_{t}}\right)/\sqrt{2\sigma_{x_{t}|w_{t}}^{2}}$ in (8), that is, $x_{t}=\mu_{x_{t}|w_{t}}+\sqrt{2\sigma_{x_{t}|w_{t}}^{2}}u_{t}$, we obtain an approximation for the integral in (8) based in (9), which yields the approximation for the log-likelihood function given by

$$\begin{array}{ccc} \ell_{a}\left(\Psi \right) & = & \sum_{t=1}^{n}\ell_{1t}\left(\xi ,\sigma_{e}^{2}\right)+\sum_{t=1}^{n}\log(\sum_{q=1}^{Q}\frac{\nu_{q}}{\sqrt{\pi }}\exp\left[\ell_{tq}\left(\mu_{tq},\phi_{tq}\right)\right]), \end{array}$$ (10)

where

$$\begin{array}{ccc} \ell_{tq}\left(\mu_{tq},\phi_{tq}\right) & = & \log\Gamma \left(\phi_{tq}\right)-\log\Gamma \left(\mu_{tq}\phi_{tq}\right)-\log\Gamma \left[\left(1-\mu_{tq}\right)\phi_{tq}\right]\\ & + & \left(\mu_{tq}\phi_{tq}-1\right)\log y_{t}+\left[\left(1-\mu_{tq}\right)\phi_{tq}-1\right]\log\left(1-y_{t}\right), \end{array}$$ (11)

here, *μ*_*tq*_ and *ϕ*_*tq*_ are the parameters of the beta distribution of *y*_*t*_ when the explanatory value *x*_*t*_ takes the value $x_{tq}^{*}=\mu_{x_{t}|w_{t}}+\sqrt{2\sigma_{x_{t}|w_{t}}^{2}}s_{q}$, $\mu_{x_{t}|w_{t}}$ and $\sigma_{x_{t}|w_{t}}^{2}$ being defined in (6) and *s*_*q*_ being the *q*-th zero of the *Q*-th order orthogonal Hermite polynomial. Consequently, from (2), we have

$$\begin{array}{c} g_{1}\left(\mu_{tq}\right)=f_{1}\left(z_{t}^{⊤},x_{tq}^{*};\alpha ,\beta \right),\;g_{2}\left(\phi_{tq}\right)=f_{2}\left(v_{t}^{⊤},x_{tq}^{*};\gamma ,\lambda \right). \end{array}$$

Typically in the beta linear models with errors in covariates the MLEa’s asymptotic distribution is normal with mean **Ψ** and covariance matrix $J_{a}^{-1}\left(\Psi \right)$ for *n* and *Q* large enough [4]. Here, we also shall consider this result to build asymptotic confidence intervals for the interest parameters of the model defined in (2) with a level *α*. We define *J*_*a*_(*θ*) as the partition of *J*_*a*_(**Ψ**) related with the interest parameters in the Appendix. Previous to *J*_*a*_(*θ*) matrix, we also present in the Appendix the score functions for the parameters of the model. Here, MLEa is the approximate maximum likelihood estimator.

### 2.2 Estimation by approximate pseudo maximum likelihood (pseudo-likelihood estimation)

This estimation method considers maximizing a function depending only of the parameters of interest, with, following [13], the nuisance parameters being replaced by consistent estimators in the original likelihood function defined in (3). Pseudo-likelihood estimation consists of first obtaining the optimal point $\hat{\xi }$ of the log-likelihood corresponding only to the nuisance parameters, which is given by $l_{r}(\xi )=\sum_{t=1}^{n}l_{1t}(\xi )$, with *ℓ*_1*t*_(*ξ*) as in (7). Once $\hat{\xi }$ is obtained, the pseudo log-likelihood is defined as $l_{\varrho }(\theta ,\hat{\xi })=\sum_{t=1}^{n}l_{1t}(\hat{\xi })+\sum_{t=1}^{n}l_{2t}(\theta ,\hat{\xi }).$ It is important to emphasize that the *ℓ*_2*t*_ integral defined in (8) will also be approximated by Gauss-Hermite quadrature. However, since $\ell_{2t}\left(\theta ,\hat{\xi }\right)$ involves only *θ*, its approximation will be a function only of the interest parameters. Let $\ell_{2t}^{†}\left(\theta ,\hat{\xi }\right)$ be the approximation for $\ell_{2t}\left(\theta ,\hat{\xi }\right)$ obtained with (9). From this, the approximate pseudo log-likelihood function for the nonlinear beta regression model with unidimensional error measures is defined as $l_{p}(\theta ,\hat{\xi })=\sum_{t=1}^{n}l_{1t}(\hat{\xi })+\sum_{t=1}^{n}l_{2t}^{†}(\theta ,\hat{\xi })$ in the form

$$\begin{array}{ccc} \ell_{p}\left(\theta ,\hat{\xi }\right) & = & -\frac{1}{2}\log\left[2\pi \left({\hat{\sigma }}_{x}^{2}+{\hat{\sigma }}_{e}^{2}\right)\right]-\frac{1}{2({\hat{\sigma }}_{x}^{2}+{\hat{\sigma }}_{e}^{2})}{\left(w_{t}-\mu_{x}\right)}^{2}\\ & + & \sum_{t=1}^{n}\log(\sum_{q=1}^{Q}\frac{\nu_{q}}{\sqrt{\pi }}\exp[\ell_{tq}\left(\ell_{tq}\left(\mu_{tq}^{\hat{r}};\phi_{tq}^{\hat{r}}\right)\right]), \end{array}$$ (12)

where *ℓ*_*tq*_(*μ*_*tq*_;*ϕ*_*tq*_), defined in (11), is evaluated at $\hat{\xi }$, that is $\mu_{tq}^{\hat{r}}=g_{1}^{-1}(f_{1}(z_{t}^{⊤}{\hat{x}}_{t}^{*};\alpha ,\beta ))$, $\phi_{tq}^{\hat{r}}=g_{2}^{-1}\left(f_{2}\left(v_{t}^{⊤},{\hat{x}}_{t}^{*};\gamma ,\lambda \right)\right)$ and ${\hat{x}}_{t}^{*}={\hat{\mu }}_{x_{t}|w_{t}}+\sqrt{2{\hat{\sigma }}_{x_{t}|w_{t}}^{2}}s_{q}$.

Let $\tilde{\theta }$ be the approximate pseudo maximum likelihood estimator of *θ*, obtained by maximizing (12). Then, under assumptions that are usually satisfied in practice, it can be shown that the asymptotic distribution of $\sqrt{n}\left(\tilde{\theta }-\theta \right)$ is normal with zero mean and covariance matrix

$$\begin{array}{ccc} \Sigma & = & I_{\theta \theta }^{-1}+I_{\theta \theta }^{-1}I_{\theta \xi }\Sigma_{\xi \xi }^{-1}I_{\theta \xi }^{⊤}I_{\theta \theta }^{-1}, \end{array}$$

with $I_{\theta \theta }=-\sum_{t=1}^{n}E\{\frac{\partial^{2}l_{pt}(\theta ,\xi )}{\partial \theta \theta^{⊤}}\}$, $I_{\theta \xi }=-\sum_{t=1}^{n}E\{\frac{\partial \ell_{pt}\left(\theta ,\xi \right)}{\partial^{2}\theta \xi^{⊤}}\}$ and $\Sigma_{\xi \xi }=\sum_{t=1}^{n}E\{\frac{\partial^{2}\ell_{rt}\left(\xi \right)}{\partial \xi \xi^{⊤}}\}$, where *ℓ*_*rt*_(*ξ*) and *ℓ*_*pt*_(*θ*,*ξ*) are, respectively, the *t*-th element of the log-likelihood restricted to perturbation parameters $l_{r}(\xi )=\sum_{t=1}^{n}l_{1t}(\xi )$, and of *ℓ*_*p*_(*θ*,*ξ*) given in (12). For the nonlinear beta regression model with measurement errors, the matrices *I*_*θ*_, *I*_*ξ*_ and *I*_*θ*_ have no explicit forms. Since the integral is approximated numerically, simply the second derivatives of *ℓ*_*rt*_(*ξ*) and *ℓ*_*en*_(*θ*,*ξ*) are used, thus the expected information matrix is replaced by the observed information matrix [14].

### 2.3 Estimation by regression calibration

When we estimate by regression calibration, the central idea is to replace the non-observed variable *x*_*t*_ by an estimate of the expected value of *x*_*t*_ given *w*_*t*_, *E*(*x*_*t*_|*w*_*t*_) in the likelihood function. If only one variable is measured with error, the calibration function is $r\left(w_{t};\xi ,\sigma_{e}^{2}\right)=\mu_{x}+k_{x}\left(w_{t}-\mu_{x}\right)$. Since $w_{t}∼N\left(\mu_{x},\sigma_{x}^{2}+\sigma_{e}^{2}\right)$, then, $\bar{w}=\sum_{t=1}^{n}w_{t}/n$ and $s_{w}^{2}=\sum_{t=1}^{n}{\left(w_{t}-\bar{w}\right)}^{2}/\left(n-1\right)$ are optimal estimators of *μ*_*x*_ and $\sigma_{x}^{2}+\sigma_{e}^{2}$, respectively. However, it is not possible to estimate $\sigma_{x}^{2}$ from the observed data *w*_1_, …, *w*_*n*_. We assume that *k*_*x*_ or $\sigma_{e}^{2}$ to be known, or, alternatively, that $\sigma_{e}^{2}$ can be estimated since it is possible to observe replicas of *w*_*t*_.

Replacing the estimated calibration function in the probability density function of *y*_*t*_ given *x*_*t*_, that is, in *f*(*y*_*t*_|*x*_*t*_;*α*, *β*, *γ*, λ) given in (1), we obtain the modified log-likelihood as

$$\ell_{rc}\left(\theta \right)=\sum_{t=1}^{n}\ell \left(\overset{ˇ}{\mu_{t}},\overset{ˇ}{\phi_{t}}\right),$$

where *ℓ*(*μ*_*t*_;*ϕ*_*t*_) = log Γ(*ϕ*_*t*_) − log Γ(*μ*_*t*_ *ϕ*_*t*_) − log Γ[(1 − *μ*_*t*_)*ϕ*_*t*_] + (*μ*_*t*_ *ϕ*_*t*_ − 1) log *y*_*t*_ + [(1 − *μ*_*t*_)*ϕ*_*t*_ − 1] log (1 − *y*_*t*_), $g\left({\overset{ˇ}{\mu }}_{tq}\right)=f_{1}\left(z_{t}^{⊤},{\overset{ˇ}{x}}_{t}^{*};\alpha ,\beta \right)$, $h\left({\overset{ˇ}{\phi }}_{tq}\right)=f_{2}\left(v_{t}^{⊤},{\overset{ˇ}{x}}_{t}^{*};\gamma ,\lambda \right)$ and ${\overset{ˇ}{x}}_{t}^{*}=\bar{w}+{\hat{k}}_{x}\left(w_{t}-\bar{w}\right)$, ${\hat{k}}_{x}$ being the known or estimated value of *k*_*x*_. Observe that the log of the modified likelihood involves only the interest parameter *θ* and is the same as that of the usual beta regression model, since $x_{t}^{*}$ plays the role of an explanatory variable that is measured with no error. Standard errors of these estimators can be estimated via nonparametric bootstrap.

## 3 Simulations

In order to check the performances of the estimation methods that were described in Section (2), we have carried out simulation studies with 10,000 Monte Carlo replications each. We consider beta regression models where some of the covariates are measured with error and, at the same time, is nonlinear with respect to the parameters associated to the covariates that are measured with no error. This was the structure we have used in our application. The model for which we perform our simulations is a particular case in the class of models we propose in Section (2).

Model parameters are estimated with the naive model, (*ι*_*naive*_), which is the traditional beta likelihood function, approximated maximum likelihood (*ι*_*a*_), approximated pseudo maximum likelihood (*ι*_*p*_) and regression calibration (*ι*_*rc*_). In all optimization processes, we have used the nonlinear BFGS quasi-Newton algorithm with numerical derivatives that is implemented in the programming language Ox [15]. Integral approximations were tried by using *Q* = 12, 30, 50 and 80 points of quadrature, these four different numbers of points yielding very similar results. We present here results for *Q* = 50.

We have made simulations for *n* = 40, 80 and 160. At each Monte Carlo replication, the *n* fixed observations of the covariate with no measurement errors, denoted by *z*_*t*1_, were obtained as independent draws from a uniform distribution on (0.2, 1.2), *t* = 1, …, *n*. Furthermore,

$$y_{t}|x_{t}∼Beta\left(\mu_{t},\phi_{t}\right),\;w_{t}=x_{t}+e_{t},\;x_{t}\overset{\text{ind}}{∼}N\left(\mu_{x},\sigma_{x}^{2}\right),\;\;e_{t}\overset{\text{ind}}{∼}N\left(0,\sigma_{e}^{2}\right),$$

*x*_*t*_ and *e*_*t*′_ being independent, *t*, *t*′ = 1, …, *n*. The simulations were performed for three different values of *k*_*x*_, namely: *k*_*x*_ = 0.50 ($\sigma_{e}^{2}=\sigma_{x}^{2}$, high measurement error), *k*_*x*_ = 0.75 ($\sigma_{e}^{2}=\sigma_{x}^{2}/3$, moderate measurement error) and *k*_*x*_ = 0.95 $\sigma_{e}^{2}=\sigma_{x}^{2}/19$, low measurement error). Finally, we consider $g_{1}\left(\mu_{t}\right)=\log\frac{\mu_{t}}{1-\mu_{t}}$, that is the logit link function and *g*_2_(*ϕ*_*t*_) = log(*ϕ*_*t*_), *t* = 1, …, *n*.

### 3.1 Scenario 1—Constant precision

The model is defined as

$$g_{1}\left(\mu_{t}\right)=\alpha_{1}+z_{t1}^{\alpha_{2}}+\beta_{1}x_{t1},\;g_{2}\left(\phi \right)=\gamma_{1},\;t=1,\ldots ,n.$$

The true values of the parameters submodels are *α*_1_ = −0.6, *α*_2_ = 2.4 and *β*_1_ = 0.8. Aiming to compare the performances of the competing estimation schemes presented in Section (2), for different precision magnitudes, we have chosen three values for *γ*_1_, namely: 2.8, 4.0 and 5.7 leading to *ϕ* = 16.44, *ϕ* = 54.60 and *ϕ* = 298.87, typically these *ϕ*’s values in beta regression represent three degree of precision model, namely: substantially low, low to medium and medium to high, respectively. Concerns to the nuisance parameters we chose *μ*_*x*_ = 0 and $\sigma_{x}^{2}=1$. Thus, for *k*_*x*_ = 0.50 ($\sigma_{e}^{2}=\sigma_{x}^{2}=1.0$, high measurement error), *k*_*x*_ = 0.75 ($\sigma_{e}^{2}=0.333$, moderate measurement error) and *k*_*x*_ = 0.95 ($\sigma_{e}^{2}=0.053$, low measurement error).

### 3.2 Scenario 2—Varying precision I

In this scenario there is a covariate measured with error in both submodels. However, the nonlinearity is only in the mean submodel, such that

$$g_{1}\left(\mu_{t}\right)=\alpha_{1}+z_{t1}^{\alpha_{2}}+\beta_{1}x_{t1},\;g_{2}\left(\phi_{t}\right)=\gamma_{1}+\lambda_{1}x_{t1},\;t=1,\ldots ,n.$$

The true values of the parameters are *α*_1_ = −0.6, *α*_2_ = 2.4, *β*_1_ = 0.8, *γ*_1_ = 2.5, λ_1_ = 0.9. In this scenario we have *μ*_*x*_ = 0 and $\sigma_{x}^{2}=1$ yielding the same values to $\sigma_{e}^{2}$ of the Scenario 1, resulting in *k*_*x*_ = 0.50 ($\sigma_{e}^{2}=1.0$), *k*_*x*_ = 0.75 ($\sigma_{e}^{2}=0.333$) and *k*_*x*_ = 0.95 ($\sigma_{e}^{2}=0.053$).

### 3.3 Scenario 3—Varying precision II

This is the most complex scenario. The beta regression model with varying precision, and the both submodels present measurement error and nonlinearity, such that the complete model is defined as

$$g_{1}\left(\mu_{t}\right)=\alpha_{1}+z_{t1}^{\alpha_{2}}+\beta_{1}x_{t1},\;g_{2}\left(\phi_{t}\right)=\gamma_{1}+v_{t1}^{\gamma_{2}}+\lambda_{1}x_{t1},\;t=1,\ldots ,n.$$

We admit that *v*_*t*1_ = *z*_*t*1_, for *t* = 1, …, *n*. The true values of the parameters are *α*_1_ = 0.7, *α*_2_ = 2.0, *β*_1_ = −1.5, *γ*_1_ = 1.5, *γ*_2_ = 2.0 and λ_1_ = 1.3. Here, *μ*_*x*_ = 1.5 and $\sigma_{x}^{2}=0.5$. Thus we have used *k*_*x*_ = 0.50, with $\sigma_{e}^{2}=0.5$, *k*_*x*_ = 0.75 with $\sigma_{e}^{2}=0.167$ and *k*_*x*_ = 0.95 with $\sigma_{e}^{2}=0.026$.

### 3.4 Simulation results

Table 1 displays the RMSE (root mean square error) of the Scenario 1’s estimators. From this table, overall, we note that the RMSE decreases considerably when the precision model, the *k*_*x*_ value and the sample size increase, which is the expected behavior.

**Table 1:** Root mean square error (RMSE) for the estimators of *α*_1_, *α*_2_, *β*_1_ and *γ*_1_ for the nonlinear beta regression model with constant precision. Scenario 1.

		$g_{1}\left(\mu_{t}\right)=\alpha_{1}+z_{t1}^{\alpha_{2}}+\beta_{1}x_{t1}$ and *g*_2_(*ϕ*) = *γ*_1_, *t* = 1, …, *n*							
*ϕ*		16.4				298.9			
		*n* = 40							
*k*_*x*_		*α*_1_	*α*_2_	*β*_1_	*γ*_1_	*α*_1_	*α*_2_	*β*_1_	*γ*_1_
0.95	*ι*_*naive*_	0.0945	0.7119	0.1051	0.2319	0.0370	0.2872	0.0613	1.0742
	*ι*_*rc*_	0.0951	0.7119	0.1027	0.2319	0.0377	0.2872	0.0411	1.0742
	*ι*_*a*_	0.0955	0.7199	0.1060	0.2982	0.0377	0.2872	0.0410	0.8217
	*ι*_*p*_	0.0955	0.7199	0.1060	0.2982	0.0377	0.2872	0.0410	0.8213
0.75	*ι*_*naive*_	0.1128	0.8215	0.2403	0.4402	0.0709	0.5379	0.2329	2.3364
	*ι*_*rc*_	0.1234	0.8215	0.1607	0.4402	0.0836	0.5379	0.1201	2.3364
	*ι*_*a*_	0.1266	0.8605	0.1862	0.8649	0.0860	0.5683	0.1112	1.5487
	*ι*_*p*_	0.1263	0.8603	0.1825	0.8310	0.0856	0.5667	0.1059	1.4792
0.50	*ι*_*naive*_	0.1312	0.9275	0.4357	0.7332	0.0970	0.7186	0.4340	2.9636
	*ι*_*rc*_	0.2887	0.9275	2.9256	0.7332	0.4810	0.7186	2.1507	2.9636
	*ι*_*a*_	0.2027	1.0201	0.4436	1.8390	0.1579	0.7832	0.3009	1.4812
	*ι*_*p*_	0.2079	1.0164	0.5249	1.6914	0.1699	0.7773	0.3853	1.3890
		*n* = 160							
*k*_*x*_		*α*_1_	*α*_2_	*β*_1_	*γ*_1_	*α*_1_	*α*_2_	*β*_1_	*γ*_1_
0.95	*ι*_*naive*_	0.0485	0.3805	0.0651	0.1411	0.0194	0.1561	0.0502	1.0949
	*ι*_*rc*_	0.0486	0.3805	0.0496	0.1411	0.0196	0.1561	0.0208	1.0949
	*ι*_*a*_	0.0487	0.3839	0.0507	0.1295	0.0195	0.1537	0.0200	0.3876
	*ι*_*p*_	0.0487	0.3839	0.0507	0.1295	0.0195	0.1537	0.0200	0.3876
0.75	*ι*_*naive*_	0.0585	0.4568	0.2275	0.4634	0.0376	0.3148	0.2286	2.3981
	*ι*_*rc*_	0.0613	0.4568	0.0736	0.4634	0.0416	0.3148	0.0582	2.3981
	*ι*_*a*_	0.0615	0.4618	0.0786	0.2301	0.0427	0.3119	0.0479	0.9346
	*ι*_*p*_	0.0615	0.4618	0.0785	0.2297	0.0426	0.3113	0.0481	0.9203
0.50	*ι*_*naive*_	0.0692	0.5462	0.4306	0.7796	0.0526	0.4500	0.4321	3.0352
	*ι*_*rc*_	0.0832	0.5462	0.1379	0.7796	0.0696	0.4500	0.1260	3.0352
	*ι*_*a*_	0.0859	0.5504	0.1676	0.7784	0.0718	0.4470	0.0904	0.9828
	*ι*_*p*_	0.0854	0.5484	0.1606	0.7149	0.0718	0.4411	0.0937	0.9565

However, this behavior does not hold for the *γ*_1_’s estimators, in special when we use the naive and regression calibration methods. It is noteworthy how the ${\hat{\gamma }}_{1}$’s RMSE for these two methods become larger when *ϕ* increases. We shall focus on *k*_*x*_ = 0.50 and *n* = 160. Be *ϕ* = 16.4 and *ϕ* = 298.9, the ${\hat{\gamma }}_{1}$’s RMSE for the naive (*ι*_*naive*_), regression calibration (*ι*_*rc*_), approximate likelihood (*ι*_*a*_) and pseudo-likelihood (*ι*_*p*_) estimation methods are (0.7796, 0.7796, 0.7784, 0.7149) and (3.0352, 3.0352, 0.9895, 0.9565), respectively. Furthermore, the RMSE of ${\hat{\gamma }}_{1}$ does not decrease for the naive and regression calibration methods when the sample size increases, notably for *k*_*x*_ = 0.50 and *k*_*x*_ = 0.75.

The best RMSE-related performances of the estimators are due the approximated likelihood schemes, overall. The largest RMSEs are of the ${\hat{\gamma }}_{1}$. When *ϕ* = 298.9 these RMSEs are larger than when *ϕ* = 16.4. However, these RMSEs decrease substantially when the sample size and reliability coefficient increase. Furthermore, the ${\hat{\alpha }}_{1}$, ${\hat{\alpha }}_{2}$ and ${\hat{\beta }}_{1}$ do not display large RMSEs when the precision is so low. Lets focus in the *ι*_*p*_ scheme, pseudo-likelihood and in the ${\hat{\beta }}_{1}$, the estimator related to measurement error. Be *k*_*x*_ = 0.75 and *n* = 40, when *ϕ* = 16.4 and *ϕ* = 298.9 their RMSEs are 0.1825 and 0.1059, respectively. Finally, it is important to note that only when *n* = 40, *k*_*x*_ = 0.50 and for the parameter *β*_1_, RMSE-related, the estimation by approximate likelihood outperforms the pseudo-likelihood method.

**Fig 1:**
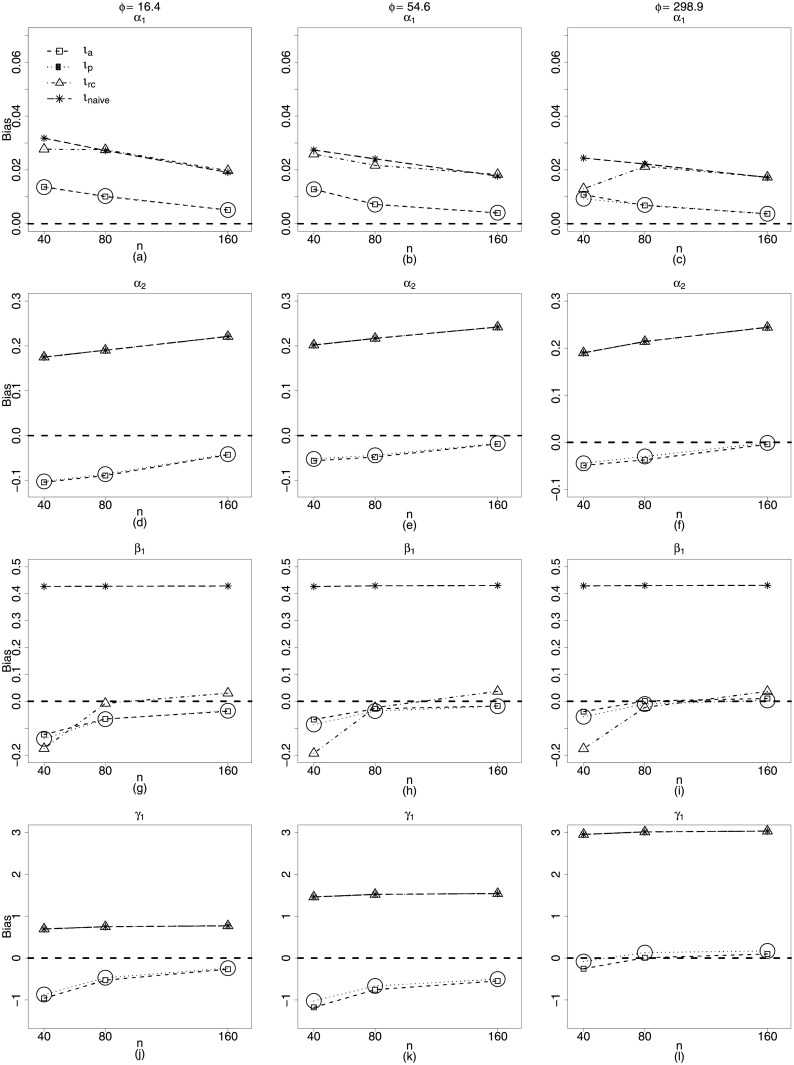
Biases of estimators of *α*_1_, *α*_2_, *β*_1_ and *γ*_1_, for *ϕ* = 16.4, 54.6, 298.9. *ι*_*a*_ (square), *ι*_*p*_ (circle), *ι*_*rc*_ (triangle) and *ι*_*naive*_ (star). **Scenario 1**: $g_{1}\left(\mu_{t}\right)=\alpha_{1}+z_{t1}^{\alpha_{2}}+\beta_{1}x_{t1}$, *g*_2_(*ϕ*) = *γ*_1_, *t* = 1, …, *n*, *n* = 40, 80, 160. **k**_**x**_ = **0.50**.

Fig 1 presents plots for the biases of the parameter estimators of the Scenario 1, when *n* = 40, 80, 160, *ϕ* ≈ 17, 55, 300 and *k*_*x*_ = 0.50. It is noteworthy as the naive structure and regression calibration perform poorly, in especial for the precision estimation. As the *ϕ* value increases, the biases of the *γ*_1_ estimators moving considerably away from zero showing absolute values close to three. Both estimation schemes also show the worst performances for estimating the nonlinearity parameter *α*_2_, since their biases moving further away from zero as the sample size increases. However, it is important to note that the absolute biases of ${\hat{\alpha }}_{2}$, equally close to 0.2. for both methods are considerably small compared to the actual value of the parameter, *α*_2_ = 2.4.

In fact, it is remarkable how badly the naive *β*_1_ estimator behaves. Its bias is constant for any value of *n* and *ϕ* and considerably high, equal to 0.4 while *β*_1_ = 0.8. Since *β*_1_ is the coefficient of the covariate measured with error, this behavior considerably disfavors the naive structure.

Regarding methods based on approximate likelihood (*ι*_*a*_ and *ι*_*p*_), the Fig 1 leads to important conclusions. In general, their biases are not markedly high, especially when the sample size increases, even when *ϕ* is quite low. As an example, for *β*_1_, the coefficient of the covariate measured with error, the largest biases of these two estimators occur when *n* = 40 and *ϕ* ≈ 17, and are close to −0.1, getting closer and closer to zero for *ϕ* ≈ 55 and *ϕ* ≈ 300 (Fig 1(g)–1(i)). Nevertheless, be *α*_2_, the nonlinearity parameter. We note that the biases of the approximate likelihood and the pseudo-likelihood estimators for this parameter are close to zero when *n* = 160 (Fig 1(d)–1(f)).

For significant value of the reliability coefficient, i.e., *k*_*x*_ = 0.50 only when the *ι*_*a*_ and *ι*_*p*_ methods are used, the biases of the estimators for all model parameters decrease as the sample size increases. and their performances are quite similar. However, for $\hat{\gamma }$, the *ι*_*p*_ method exhibits slightly lower bias than the *ι*_*p*_ method, particularly for *n* = 40.

The fact described above is the first evidence of a better performance of the *ι*_*p*_ method, typically. The smaller the $\hat{\gamma }$’s bias, the smaller the bias of the variances of the model estimators and the better the performance of the confidence intervals and hypothesis tests related to the model parameters.

In what follows we shall discuss estimation by confidence interval with respect to Scenario 1. The Table 2 displays the coverage rates of the estimated asymptotically 95% confidence intervals for *k*_*x*_ = 0.50, 0.75, 0.95, *n* = 80 and *ϕ* = 16.4, 298.9.

**Table 2:** Coverage rates and average lengths of the nominal 95% confidence interval estimators. *k*_*x*_ = 0.50, 0.75, 0.95. **Scenario 1**: $g_{1}\left(\mu_{t}\right)=\alpha_{1}+z_{t1}^{\alpha_{2}}+\beta_{1}x_{t1}$, *g*_2_(*ϕ*) = *γ*_1_, *t* = 1, …, *n*, **n = 80**.

Parameters	Interval	*k*_*x*_ = 0.95				*k*_*x*_ = 0.75				*k*_*x*_ = 0.50			
		inf	right	sup	length	inf	right	sup	length	inf	right	sup	length
		*ϕ* = 16.4											
*α*_1_	*ι*_*naive*_	2.59	94.18	3.23	0.28	2.13	93.92	3.95	0.33	1.73	93.67	4.60	0.38
	*ι*_*rc*_	2.65	93.43	3.92	0.28	2.78	91.88	5.34	0.34	4.47	86.79	8.74	0.39
	*ι*_*a*_	2.61	93.43	2.99	0.28	2.45	94.49	3.06	0.35	2.55	94.06	3.39	0.49
	*ι*_*p*_	2.94	93.43	3.59	0.27	2.79	93.81	3.40	0.35	2.16	94.71	3.13	0.87
*α*_2_	*ι*_*naive*_	2.14	92.02	5.84	2.23	1.61	90.12	8.27	2.55	1.29	87.30	11.41	2.83
	*ι*_*rc*_	3.24	90.46	6.30	2.25	2.34	88.94	8.72	2.59	1.70	87.50	10.80	2.89
	*ι*_*a*_	2.38	92.38	5.24	2.25	2.68	91.65	5.67	2.67	6.97	84.68	8.35	2.90
	*ι*_*p*_	3.55	90.41	6.04	2.19	3.92	89.45	6.63	2.62	5.92	86.39	7.69	4.36
*β*_1_	*ι*_*naive*_	0.43	88.45	11.12	0.26	0.00	9.50	90.50	0.26	0.00	0.00	100.00	0.23
	*ι*_*rc*_	3.50	91.87	4.63	0.26	4.49	83.77	11.74	0.30	11.12	66.59	22.29	0.40
	*ι*_*a*_	3.07	94.05	2.88	0.28	2.18	95.00	2.82	0.43	10.63	85.23	4.14	0.75
	*ι*_*p*_	3.40	93.46	3.14	0.27	2.54	94.25	3.21	0.43	4.36	91.10	4.54	2.02
*γ*_1_	*ι*_*naive*_	1.28	92.08	6.64	0.60	0.00	20.94	79.06	0.60	0.00	0.28	99.72	0.59
	*ι*_*rc*_	1.84	90.61	7.55	0.61	0.00	22.78	77.22	0.61	0.00	0.56	99.44	0.61
	*ι*_*a*_	5.24	93.60	1.16	0.70	0.82	97.39	1.79	1.28	10.63	85.69	3.68	2.49
	*ι*_*p*_	6.27	92.16	1.57	0.68	1.91	95.93	2.16	1.30	8.86	86.96	4.18	10.61
		*ϕ* = 298.9											
*α*_1_	*ι*_*naive*_	2.34	94.42	3.24	0.11	2.01	93.95	4.04	0.21	1.71	93.83	4.46	0.29
	*ι*_*rc*_	2.79	93.22	3.99	0.11	3.55	90.38	6.07	0.22	6.02	83.24	10.74	0.30
	*ι*_*a*_	2.80	94.39	2.81	0.11	7.79	84.62	7.59	0.20	5.76	87.34	6.90	0.34
	*ι*_*p*_	2.87	93.87	3.26	0.11	6.11	87.72	6.17	0.26	3.03	92.76	4.21	0.93
*α*_2_	*ι*_*naive*_	2.08	93.37	4.55	0.92	1.28	90.93	7.79	1.69	0.97	87.91	11.12	2.19
	*ι*_*rc*_	2.60	91.96	5.44	0.93	1.98	89.82	8.20	1.71	1.48	87.26	11.26	2.24
	*ι*_*a*_	2.93	93.32	3.75	0.92	13.91	72.78	13.31	1.33	19.73	61.40	18.87	1.47
	*ι*_*p*_	3.81	91.81	4.38	0.89	12.72	75.20	12.08	1.57	12.08	75.29	12.63	4.07
*β*_1_	*ι*_*naive*_	0.00	56.37	43.63	0.10	0.00	0.14	99.86	0.17	0.00	0.00	100.00	0.18
	*ι*_*rc*_	1.95	92.34	5.71	0.10	2.99	81.55	15.46	0.22	10.09	64.87	25.04	0.37
	*ι*_*a*_	2.39	95.27	2.34	0.11	8.28	85.77	5.95	0.23	15.00	73.27	11.73	0.36
	*ι*_*p*_	2.79	94.52	2.69	0.11	4.12	90.92	4.96	0.31	0.60	93.38	6.02	2.12
*γ*_1_	*ι*_*naive*_	0.00	0.00	100.00	0.62	0.00	0.00	100.00	0.61	0.00	0.00	100.00	0.60
	*ι*_*rc*_	0.00	0.00	100.00	0.62	0.00	0.00	100.00	0.62	0.00	0.00	100.00	0.60
	*ι*_*a*_	0.60	97.20	2.20	2.38	36.37	56.13	7.50	3.14	48.92	39.38	11.70	2.80
	*ι*_*p*_	1.26	95.96	2.78	2.31	33.86	58.07	8.07	4.14	29.86	57.34	12.80	12.64

At this point, it is interesting to note how the high ${\hat{\gamma }}_{1}$ biases negatively affected the performance of the interval estimation. In particular, the considerable overestimation of *γ*_1_ when we use the naive and regression calibration methods leads to variances of the estimators highly underestimated. Especially the larger the value of *ϕ*. Therefore, we have interval lengths that are too small, to the point of not coverage the true values of the parameters. For example, when *ϕ* = 298.8 the coverage rates for the parameter *γ*_1_ based on the two methods are equal to zero, and 100% of the estimated values are greater than the true value of the parameter (Table 2).

We turn now to the coefficient for the covariate with measurement error, i.e., the parameter *β*_1_. Since ${\hat{\beta }}_{1}$ relative to the naive method considerably overestimates that parameter, regardless of the value of *ϕ* (Fig 1(j)–1(l)), in association with the underestimation of variances already commented above, its *β*_1_ interval estimator produces values that are 100% higher than the true value of the parameter. The performance of the interval estimator based on the regression calibration method is considerably better than the naive related to *β*_1_. However, it is still considerably poorer than that of the *ι*_*a*_ and *ι*_*b*_ methods. For *k*_*x*_ = 0.50 and *ϕ* = 298.9, the coverage rates and average lengths for *ι*_*rc*_, *ι*_*a*_ and *ι*_*p*_ are (66.59%, 73.27%, 93.38%) and (0.40, 0.75, 2.02), respectively. Here we note the outperformance of the *ι*_*p*_ scheme. Its coverage rate is the closest the nominal level 95% and the average length equal to 2.02 decreases to 0.31, for *k*_*x*_ = 0.75, ensuring a coverage rate closer to the nominal level (Table 2).

We shall now evaluate the interval estimators of *α*_2_. Based on the Table 2 we note that for *ϕ* = 16.4, the schemes: *ι*_*naive*_, *ι*_*rc*_, *ι*_*a*_ and *ι*_*p*_ show coverage rates near each other and equal to (90.12%, 88.94%, 91.65%, 89.45%), respectively, for *k*_*x*_ = 0.75. These coverage rates are still closer when *k*_*x*_ = 0.50 and *k*_*x*_ = 0.95. However, when *ϕ* = 298.9 the behavior of the four methods differs considerably. When *k*_*x*_ = 0.75 those coverage rates above become equal to (90.93%, 89.82%, 72.78%, 75.20%). When *k*_*x*_ = 0.50, these coverage rates differ further, in especial related to *ι*_*a*_ estimator, being equal to (87.91%, 87.26%, 61.40%, 75.29%). Here it is necessary to increase the sample size to better understand the behavior of the estimators.

**Fig 2:**
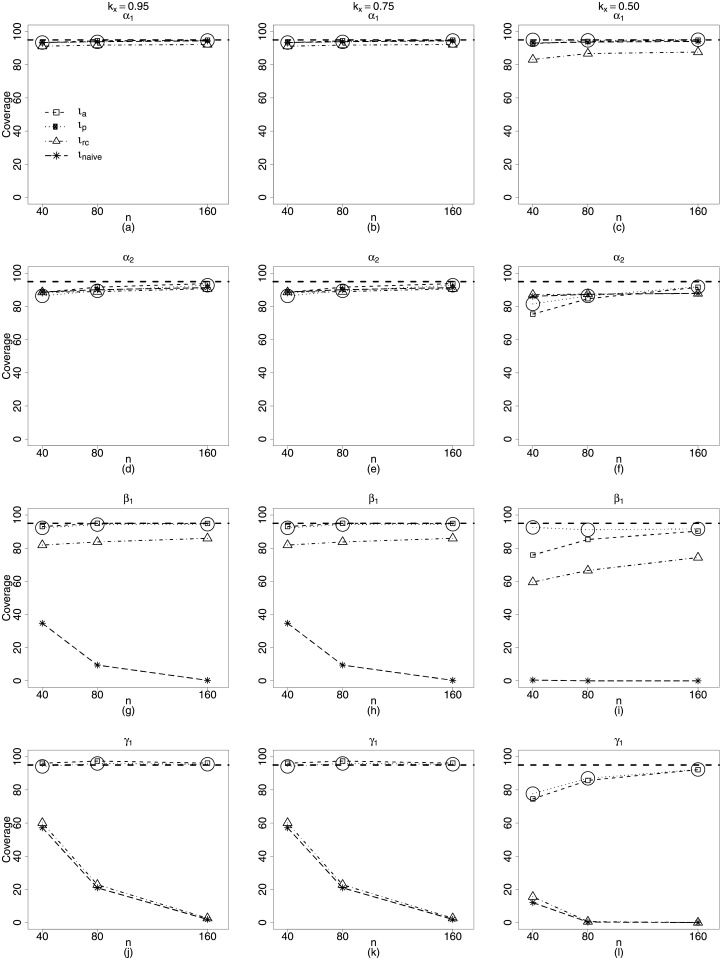
Coverage rates of the nominal 95% confidence intervals for the parameters *α*_1_, *α*_2_, *β*_1_ and *γ*_1_. *k*_*x*_ = 0.95, 0.75, 0.50. **Scenario 1**: $g_{1}\left(\mu_{t}\right)=\alpha_{1}+z_{t1}^{\alpha_{2}}+\beta_{1}x_{t1}$, *g*_2_(*ϕ*) = *γ*_1_, *t* = 1, …, *n*. *n* = 40, 80, 160 *ϕ* = **16.4**.

Thus, Fig 2 displays the coverage rates of the interval estimators of the beta regression model with measurement errors and nonlinearity with constant precision equal to 16.4. We consider *n* = 40, 80, 160 and *k*_*x*_ = 0.95, 0.75, 0.50.

Those results of coverage rates are related to the biases of the point estimators. In this case, the *ι*_*naive*_ and *ι*_*rc*_ methods have been incorrectly favored for being more biased. Their respective point estimators slightly overestimate *α*_2_, this fact associate with the considerably underestimate the variance, in particular when *ϕ* = 298.9 (Fig 1(l)) leads to interval estimators that cover the true value of the parameters without loss of accuracy. However, this fact changes as the sample size increases. We can see in Fig 2(f) that when *n* = 160, the coverage rates of the interval estimators for *α*_2_, related to *ι*_*a*_ and *ι*_*p*_ methods, are close to the nominal level of 95%, while those of the *ι*_*naive*_ and *ι*_*rc*_ methods remain constant for all sample sizes and close to 90%.

The *ι*_*a*_ method is the most illustrative and true in its performance, even for *n* = 80 and *k*_*x*_ = 0.50 (*ϕ* = 298.9). The average bias of its respective ${\hat{\gamma }}_{1}$ can be considered equal to zero. Besides, its largest ${\hat{\alpha }}_{2}$ average bias is less than −0.1 while *α*_2_ = 2.4. Thus, the chance that their interval estimates cover the true value of the parameter only depend on the goodness of approximation of the point estimator’s distribution to the normal distribution. For the *ι*_*a*_ estimators this approximation seems to improve as the sample size increases, once that coverage rate equal to 84.68% cited above, for *n* = 80 and *ϕ* = 16.4, increases and remains close to the nominal level, in this case 95%. This behavior can be seen in the Fig 2(f).

From this figure, we confirm what we had discussed in the last paragraph. The interval estimators of the *ι*_*a*_ and *ι*_*p*_ methods are the ones that present the closest coverage rates to the nominal level of 95%, taking into account all parameters of the model. Most importantly, these rates get closer to the nominal level when the sample size increases. For the nonlinear beta regression with measurement error and constant precision, we should conclude that the best performance regarding interval estimation, concerns the approximate pseudo maximum likelihood method.

Table 3 presents the RMSE (root mean square error) of estimators for second and third scenarios. We begin by considering the second scenario. We observe that, in practically all situations, this RMSE decreases when the sample size increases. The exceptions lie in the estimation of *γ*_1_ of the precision model. When we use the naive model and regression calibration with significant values of the reliability coefficient *k*_*x*_, i.e., *k*_*x*_ = 0.75 and *k*_*x*_ = 0.50. For example, when using the naive model for *k*_*x*_ = 0.50, we obtained $\text{RMSE}\left({\hat{\gamma }}_{1}\right)=\left(0.6956,0.7475,0.7660\right)$, respectively, for *n* = (40, 80, 160).

**Table 3:** Root mean square error (RMSE) for the estimators of *α*_1_, *α*_2_, *β*_1_, *γ*_1_, *γ*_2_ and λ_1_ for the nonlinear beta regression model with nonconstant precision. Scenario 2 and Scenario 3.

			$g_{1}\left(\mu_{t}\right)=\alpha_{1}+z_{t1}^{\alpha_{2}}+\beta_{1}x_{t1}$ and *g*_2_(*ϕ*_*t*_) = *γ*_1_ + λ_1_ *x*_*t*1_					$g_{1}\left(\mu_{t}\right)=\alpha_{1}+z_{t1}^{\alpha_{2}}+\beta_{1}x_{t1}$ and $g_{2}(\phi_{t})=\gamma_{1}+v_{t1}^{\gamma_{2}}+\lambda_{1}x_{t1}$,					
*k*_*x*_	*n*		*α*_1_	*α*_2_	*β*_1_	*γ*_1_	λ_1_	*α*_1_	*α*_2_	*β*_1_	*γ*_1_	*γ*_2_	λ_1_
0.95	40	*ι*_*naive*_	0.1132	0.7682	0.1163	0.2426	0.2903	0.2042	0.4090	0.1247	0.4926	1.2504	0.4405
		*ι*_*rc*_	0.1137	0.7682	0.1123	0.2433	0.2955	0.1483	0.4090	0.0825	0.5342	1.2504	0.4149
		*ι*_*a*_	0.1138	0.7717	0.1135	0.3348	0.3141	0.1569	0.4231	0.0878	0.8864	1.3151	0.5074
		*ι*_*p*_	0.1138	0.7717	0.1135	0.3348	0.3141	0.1568	0.4231	0.0877	0.8856	1.3140	0.5069
	80	*ι*_*naive*_	0.0858	0.6358	0.0867	0.1687	0.2162	0.1847	0.3510	0.1160	0.4179	1.2012	0.4039
		*ι*_*rc*_	0.0861	0.6358	0.0742	0.1691	0.2015	0.1250	0.3510	0.0705	0.4620	1.2012	0.3730
		*ι*_*a*_	0.0858	0.6377	0.0746	0.2042	0.1979	0.1261	0.3646	0.0701	0.6522	1.1742	0.4113
		*ι*_*p*_	0.0858	0.6377	0.0746	0.2042	0.1979	0.1260	0.3646	0.0700	0.6518	1.1736	0.4111
	160	*ι*_*naive*_	0.0581	0.4101	0.0723	0.1368	0.1857	0.1567	0.2457	0.1030	0.2996	1.0786	0.3632
		*ι*_*rc*_	0.0583	0.4101	0.0528	0.1374	0.1592	0.0879	0.2457	0.0505	0.3489	1.0786	0.3225
		*ι*_*a*_	0.0585	0.4122	0.0530	0.1343	0.1381	0.0877	0.2611	0.0488	0.3965	0.9533	0.2649
		*ι*_*p*_	0.0585	0.4122	0.0530	0.1343	0.1381	0.0877	0.2611	0.0488	0.3965	0.9529	0.2649
0.75	40	*ι*_*naive*_	0.1340	0.8891	0.2553	0.4274	0.4763	0.6845	0.6917	0.4640	0.6493	1.6562	0.7947
		*ι*_*rc*_	0.1430	0.8893	0.1803	0.4352	0.4232	0.3143	0.6917	0.1964	0.9770	1.6562	0.6683
		*ι*_*a*_	0.1409	0.9013	0.1836	0.7116	0.5336	0.3219	0.8580	0.1938	2.1235	2.2013	1.0610
		*ι*_*p*_	0.1404	0.9006	0.1799	0.6811	0.5071	0.3113	0.8562	0.1855	2.0538	2.1559	1.0282
	80	*ι*_*naive*_	0.1038	0.7448	0.2397	0.4547	0.4547	0.6668	0.6070	0.4563	0.6135	1.6753	0.7780
		*ι*_*rc*_	0.1094	0.7448	0.1154	0.4594	0.3353	0.2680	0.6070	0.1699	0.9286	1.6753	0.6339
		*ι*_*a*_	0.1035	0.7381	0.1173	0.3801	0.3202	0.2757	0.8135	0.1654	1.7196	2.1238	0.8920
		*ι*_*p*_	0.1035	0.7380	0.1166	0.3745	0.3130	0.2673	0.8126	0.1593	1.6758	2.0838	0.8670
	160	*ι*_*naive*_	0.0705	0.5000	0.2347	0.4661	0.4480	0.6412	0.4455	0.4473	0.5971	1.6298	0.7493
		*ι*_*rc*_	0.0749	0.5000	0.0802	0.4703	0.3052	0.1878	0.4455	0.1283	0.8971	1.6298	0.5794
		*ι*_*a*_	0.0702	0.4840	0.0797	0.2170	0.2022	0.1844	0.6788	0.1107	0.9473	1.8018	0.5794
		*ι*_*p*_	0.0703	0.4840	0.0798	0.2174	0.2015	0.1813	0.6784	0.1085	0.9265	1.7809	0.5679
0.50	40	*ι*_*naive*_	0.1594	1.0040	0.4475	0.6956	0.6656	1.2176	0.8865	0.8412	0.8002	1.7773	1.0005
		*ι*_*rc*_	1.1022	1.0040	4.0169	2.2007	7.5492	0.8632	0.8865	0.5608	1.6009	1.7772	0.8471
		*ι*_*a*_	0.2124	1.0420	0.4163	1.3432	0.9974	0.5995	1.0594	0.3769	2.0546	2.3897	1.1270
		*ι*_*p*_	0.2259	1.0317	0.5150	1.2253	0.9892	0.5769	1.0520	0.3627	1.9312	2.3062	1.0705
	80	*ι*_*naive*_	0.1276	0.8540	0.4410	0.7475	0.6615	1.2049	0.7907	0.8355	0.7923	1.8151	0.9887
		*ι*_*rc*_	0.1557	0.8540	0.2777	0.7582	0.4910	0.6787	0.7907	0.4412	1.4915	1.8151	0.7611
		*ι*_*a*_	0.1389	0.8471	0.2259	0.7885	0.5571	0.4929	0.9886	0.3088	1.7557	2.3761	0.9814
		*ι*_*p*_	0.1391	0.8443	0.2337	0.7464	0.5162	0.4861	0.9837	0.3032	1.6658	2.2847	0.9384
	160	*ι*_*naive*_	0.0891	0.6079	0.4388	0.7660	0.6598	1.1822	0.6115	0.8312	0.8025	1.7750	0.9793
		*ι*_*rc*_	0.1065	0.6079	0.1521	0.7737	0.4313	0.3725	0.6115	0.5561	1.3753	1.7750	0.6858
		*ι*_*a*_	0.0913	0.5566	0.1456	0.4936	0.3439	0.3167	0.8012	0.2008	1.1880	2.0265	0.6806
		*ι*_*p*_	0.0915	0.5557	0.1470	0.4785	0.3287	0.3085	0.8004	0.1953	1.1401	1.9649	0.6557

In fact, for smaller values of *k*_*x*_ it is reasonable that for at least one estimator (in our case it was ${\hat{\gamma }}_{1}$) we should obtain a larger RMSE when *n* increases when running the naive model. This is because, for small values of *k*_*x*_, the naive model represents a poor approximation for the true model and larger values of *n* represent more information about data. Therefore, larger values of *n* will detect the approximation is not good. This is translated in a larger RMSE for at least one estimator. Also, since regression calibration does not work with the full information about data distribution, but only with moments, it is expected that this loss of information that is intrinsic to regression calibration will be felt in the estimation process if more data are available. Anyway, the poor performance of the naive model is a clear indication of the necessity, in practice, of the model here proposed.

We turn now to the parameter *β*_1_, which is the coefficient for the covariate that has error measurement. Estimation by approximate maximum likelihood (*ι*_*a*_) and approximate pseudo maximum likelihood (*ι*_*p*_) present better performances. This is expected, since they use the full information about data distribution. We then investigate if calibration regression has comparable performance, and we can see it does not. For example, in the first scenario, for *k*_*x*_ = 0.50 and *n* = 40, we have for the ${\hat{\lambda }}_{1}$ estimator that $\text{RMSE}({\hat{\lambda }}_{1})=7.549$, while the values of the RMSE are typically smaller than 1 for the other estimators.

In general, with respect to the complete estimation of the model, when we have large measurement errors, *k*_*x*_ = 0.50, and the sample size increases, the performances of the approximate pseudo maximum likelihood and the approximate maximum likelihood are comparable. Therefore, the pseudo maximum likelihood method is here recommended.

From the simulation results in Table 3, we can compare, for different reliability coefficients, the performances of the different estimators in terms of RMSE for the beta regression model with measurement errors in one covariate and nonlinearity in one parameter both for mean and precision submodel, i.e., **Scenario 3**. For the coefficient *β*_1_ of the covariate with measurement error, the naive model is the one presenting the worst performance, in particular, when the reliability coefficient is *k* = 0.50. For example, for *n* = 80 and *k* = 0.50, we have the RMSEs of the estimators *ι*_*naive*_ *ι*_*rc*_, *ι*_*a*_ and *ι*_*p*_ for *β*_1_ given, respectively, by 0.8355, 0.4412, 0.3088 and 0.3032. Thus, the approximate maximum likelihood and approximate pseudo maximum likelihood estimators present much better performances. The much worst performance of the naive estimator indicates again the usefulness of our proposed model. Also, we observe that the approximate maximum likelihood and approximate pseudo maximum likelihood are comparable, which means that pseudo-likelihood can be a useful tool in our specific proposal.

**Fig 3:**
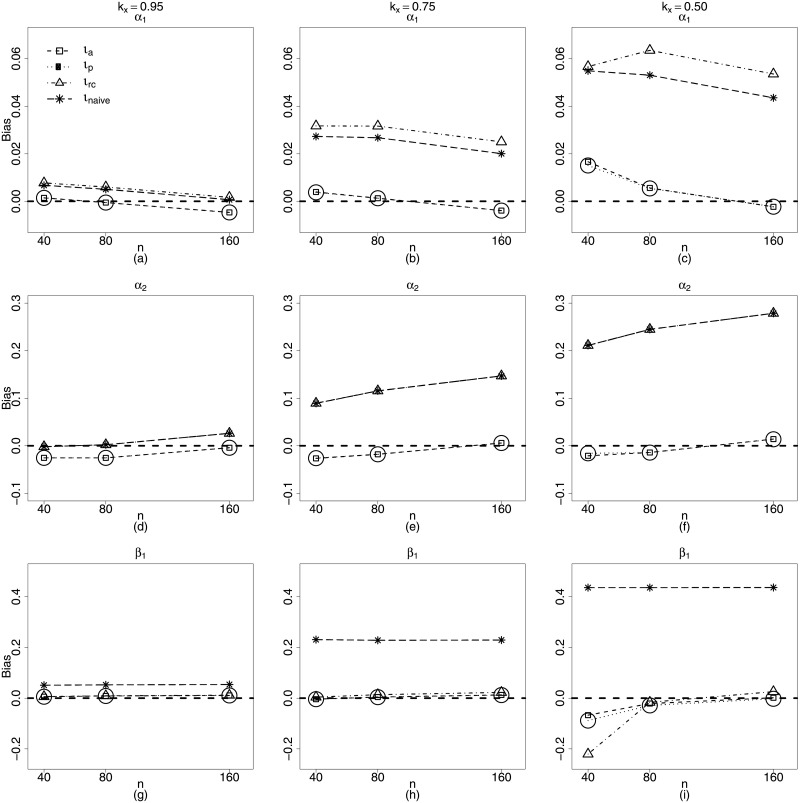
Biases of estimators of *α*_1_, *α*_2_ and *β*_1_ for *k*_*x*_ = 0.95, 0.75, 0.50. *ι*_*a*_ (square), *ι*_*p*_ (circle), *ι*_*rc*_ (triangle) and *ι*_*naive*_ (star). **Scenario 2**: $g_{1}\left(\mu_{t}\right)=\alpha_{1}+z_{t1}^{\alpha_{2}}+\beta_{1}x_{t1}$, *g*_2_(*ϕ*_*t*_) = *γ*_1_ + λ_1_ *x*_*t*1_, *t* = 1, …, *n*, *n* = 40, 80, 160.

**Fig 4:**
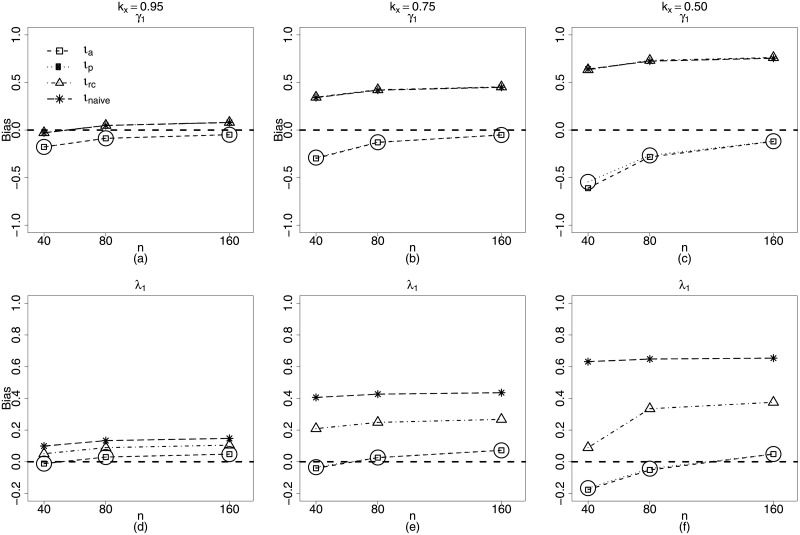
Biases of estimators of *γ*_1_ and λ_1_ for *k*_*x*_ = 0.95, 0.75, 0.50. *ι*_*a*_ (square), *ι*_*p*_ (circle), *ι*_*rc*_ (triangle) and *ι*_*naive*_ (star). **Scenario 2**: $g_{1}\left(\mu_{t}\right)=\alpha_{1}+z_{t1}^{\alpha_{2}}+\beta_{1}x_{t1}$, *g*_2_(*ϕ*_*t*_) = *γ*_1_ + λ_1_ *x*_*t*1_, *t* = 1, …, *n*, *n* = 40, 80, 160.

Figs 3 and 4 present plots for the biases of the estimators of the parameters in Scenario 2, for *n* = 40, 80, 160. It is possible to see that the naive structure and regression calibration present much worst biases. Not only that, but it was observed for these two last estimators, that absolute values of biases increase with measurement error. In particular, consider ${\hat{\alpha }}_{2}$ and ${\hat{\beta }}_{1}$, estimators we are very interested in, since they are related with the nonlinearity and measurement error of the mean submodel. Fig 3(d)–3(i) show how biases of both estimators are near zero when we use approximate likelihood (*ι*_*a*_) and approximate pseudo likelihood (*ι*_*p*_) for all sample sizes.

It is remarkable how bias of ${\hat{\alpha }}_{2}$, (related to nonlinearity) increases with measurement error for the regression calibration method. (Fig 3(d)–3(f)). This same behavior can be observed for the precision submodel parameters, in particular for ${\hat{\lambda }}_{1}$, which estimates the coefficient of the covariate with measurement error. In this case, the biases of the estimators using regression calibration increase considerably for large sample sizes and measurement errors. (Fig 4(d)–4(f)). In fact only approximate maximum likelihood and approximate pseudo maximum likelihood produce estimators with biases tending to zero as the sample size increases.

**Fig 5:**
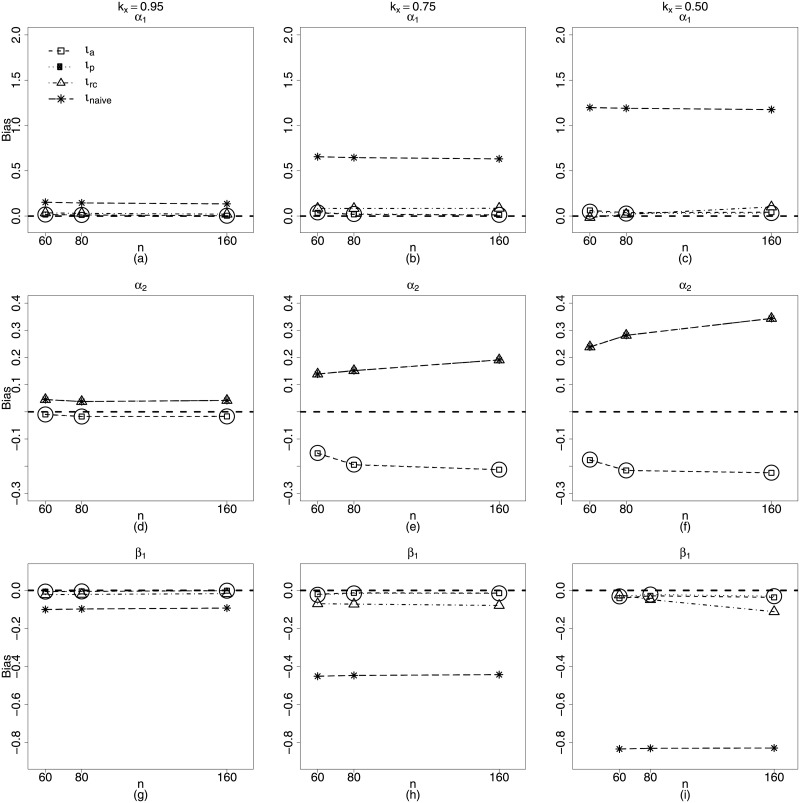
Biases of estimators of *α*_1_, *α*_2_ and *β*_1_ for *k*_*x*_ = 0.95, 0.75, 0.50. *ι*_*a*_ (square), *ι*_*p*_ (circle), *ι*_*rc*_ (triangle) and *ι*_*naive*_ (star). **Scenario 3**: $g_{1}\left(\mu_{t}\right)=\alpha_{1}+z_{t1}^{\alpha_{2}}+\beta_{1}x_{t1}$, $g_{2}\left(\phi_{t}\right)=\gamma_{1}+v_{t}^{\gamma_{2}}+\lambda_{1}x_{t1}$, *t* = 1, …, *n*, *n* = 40, 80, 160.

**Fig 6:**
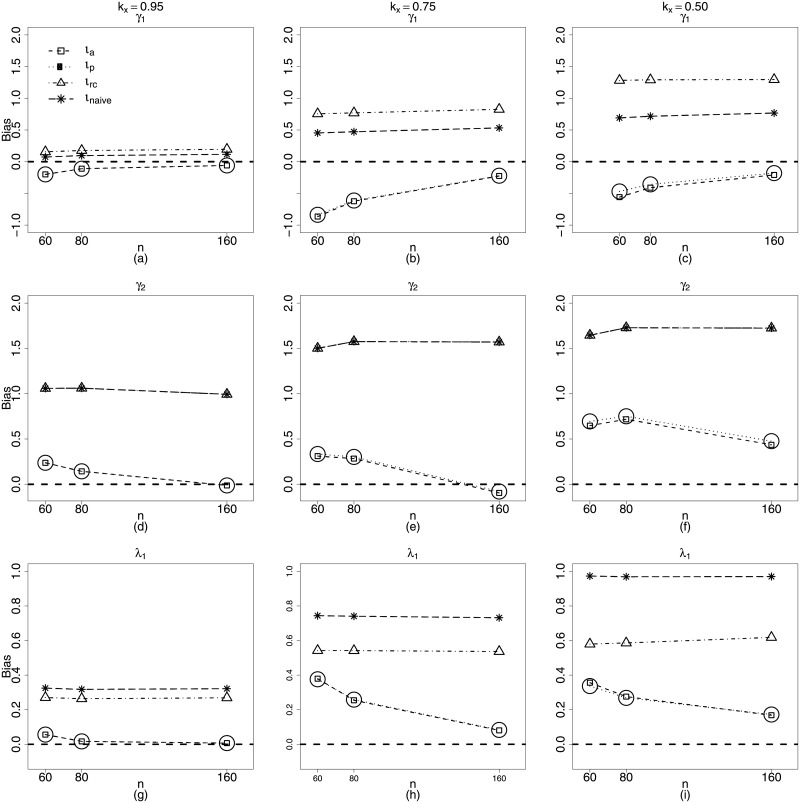
Biases of estimators of *γ*_1_, *γ*_2_ and λ_1_ for *k*_*x*_ = 0.95, 0.75, 0.50. *ι*_*a*_ (square), *ι*_*p*_ (circle), *ι*_*rc*_ (triangle) and *ι*_*naive*_ (star). **Scenario 3**: $g_{1}\left(\mu_{t}\right)=\alpha_{1}+z_{t1}^{\alpha_{2}}+\beta_{1}x_{t1}$, $g_{2}\left(\phi_{t}\right)=\gamma_{1}+v_{t}^{\gamma_{2}}+\lambda_{1}x_{t1}$, *t* = 1, …, *n*, *n* = 40, 80, 160.

Figs 5 and 6 present the plots of biases of the estimators of the parameters in **Scenario 3**, namely: nonlinear predictors with measurement error on both submodels. Approximate maximum likelihood (*ι*_*a*_) and approximate pseudo maximum likelihood (*ι*_*p*_) present much better performances than those of naive and regression calibration methods (*ι*_*rc*_) for ${\hat{\beta }}_{1}$, the estimator of the coefficient of the covariate with measurement error (mean submodel). (Fig 5(g)–5(i)). Estimators of the parameters related to nonlinearity are more biased, but, even so, the performances of *ι*_*a*_ and *ι*_*p*_ methods are slightly superior to those of the *ι*_*Naive*_ and *ι*_*rc*_, in particular for larger values of the measurement error (Fig 5(f)). We can also check that, for the parameters of the precision submodel, the maximum likelihood based methods are clearly superior (Fig 6). In fact, once more, only the *ι*_*a*_ and *ι*_*p*_ methods yield estimators with biases decreasing when sample size increases.

**Fig 7:**
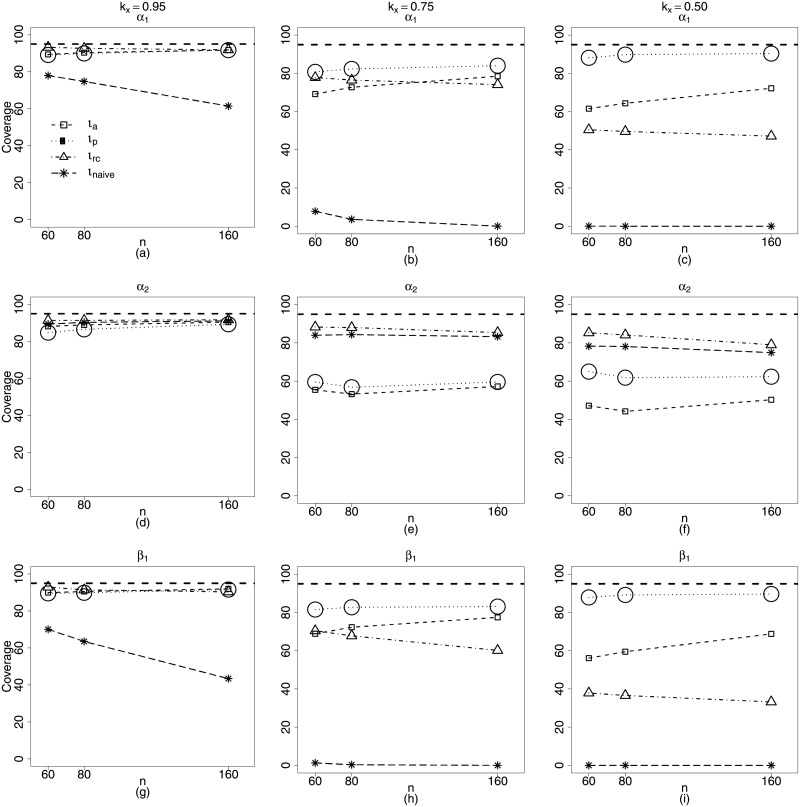
Coverage rates of the nominal 95% confidence intervals for the parameters *α*_1_, *α*_2_ and *β*_1_. *ι*_*a*_ (square), *ι*_*p*_ (circle), *ι*_*rc*_ (triangle) and *ι*_*naive*_ (star). **Scenario 3**: $g_{1}\left(\mu_{t}\right)=\alpha_{1}+z_{t1}^{\alpha_{2}}+\beta_{1}x_{t1}$, $g_{2}\left(\phi_{t}\right)=\gamma_{1}+v_{t}^{\gamma_{2}}+\lambda_{1}x_{t1}$, *t* = 1, …, *n*, *n* = 40, 80, 160.

**Fig 8:**
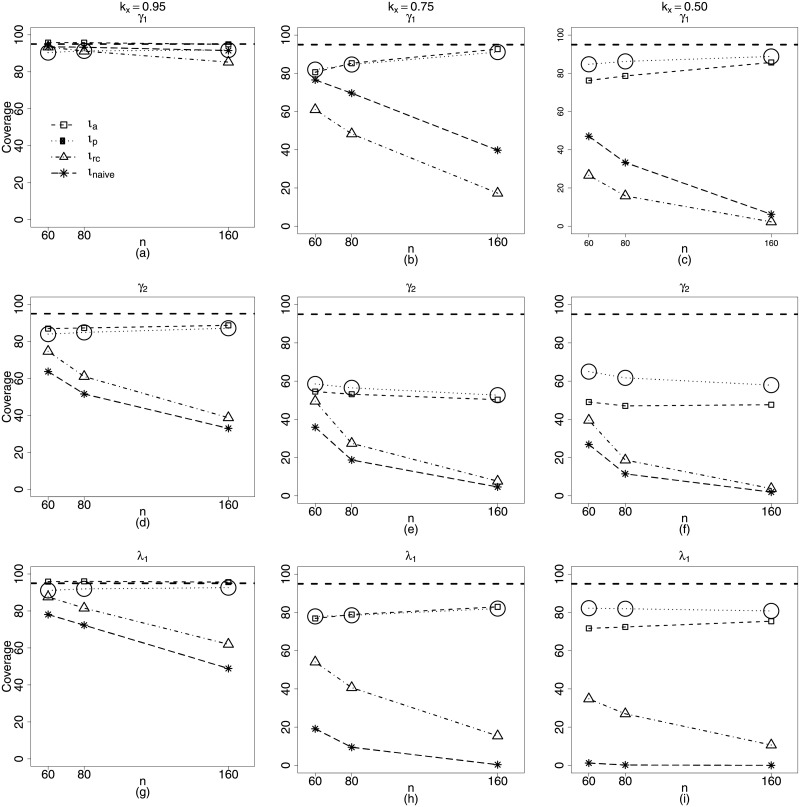
Coverage rates of the nominal 95% confidence intervals for the parameters *γ*_1_, *γ*_2_ and λ_1_. *ι*_*a*_ (square), *ι*_*p*_ (circle), *ι*_*rc*_ (triangle) and *ι*_*naive*_ (star). **Scenario 3**: $g_{1}\left(\mu_{t}\right)=\alpha_{1}+z_{t1}^{\alpha_{2}}+\beta_{1}x_{t1}$, $g_{2}\left(\phi_{t}\right)=\gamma_{1}+v_{t}^{\gamma_{2}}+\lambda_{1}x_{t1}$, *t* = 1, …, *n*, *n* = 40, 80, 160.

Figs 7 and 8 present the coverage rates for the different sample sizes and reliability coefficients of the interval estimators of the beta regression model with measurement errors and nonlinearity in both submodels (Scenario 3). From those figures, it becomes evident how the approximate pseudo maximum likelihood method is a good alternative to estimate the parameters of the models.

It is still important to stress how inference based on interval estimation for *β*_1_ can be affected when we do not consider that the covariate is measured with error, that is, when we use the naive method. That becomes more remarkable if we think this is perhaps the parameter we are more interested in, the coefficient of the covariate in the mean submodel. Fig 7(g)–7(i) clearly shows how we lose with the naive estimation, even when the measurement error is small, *k*_*x*_ = 0.95, since coverage rates for the naive estimation become considerably distant from the nominal 95% level. This is particular true for large sample sizes (Fig 7(g)). The sample size *n* = 160 is information enough to detect that we may be dealing with a wrong asymptotic normal distribution for the parameters, or, perhaps, even with nonconsistent parameters, due to the wrong assumption that the covariate has no measurement error. This behavior tends to become more seriously large as the measurement error increases, as expected (Fig 7(h) and 7(i)).

Fig 8 presents interval estimation of the parameters of the precision submodel. Here we emphasize, in particular, the very good performance of approximate pseudo maximum likelihood estimation. Considering all parameters, measurement errors and sample sizes, this seems to be the recommended method. This is an important aspect, since good estimation of the precision parameters of the observations is directly linked with the efficiency of the estimators and robustness of hypotheses tests about the model parameters.

## 4 An application: Fluid Catalytic Cracking (FCC)

Fluid Catalytic Cracking (FCC) is an important chemical process used in petroleum refineries to convert heavy hydrocarbons in small molecules with high comercial value. This process is accomplished by contact of those hydrocarbons with a catalyst having as main component a mineral that is known as zeolite Y [16]. A chemical element that can be found in FCC is vanadium. This chemical element takes part in the process by reducing, among other characteristics, the crystallinity of zeolite Y, in particular when water steam is present. The chemical reaction also depends on temperature, which must be near 720 °*C* (Salazar, 2005). Hence, at the end of the process, it is important to study how crystallinity fraction of zeolite Y is influenced by different concentrations of vanadium, by water steam and by temperature. The data set is constituted of *n* = 28 observations. The authors report that the response is moderately concentrated in the upper part of the unit interval. According to them, 75% of the observations are not smaller than 0.77. This characteristic will be considered as an important factor in our modeling.

[5] have modeled the response, considering the beta and simplex distributions, by using a nonlinear predictor for the mean but without measurement errors in the covariates. However, following [16], the concentration of vanadium for each observation was determined by a spectrophotometric method, with the aid of a chemical complex that includes the element and using a calibration curve. This calibration curve was defined with the same spectrophotometric method, by using another complex, that includes vanadium and a different reagent. Therefore, the concentration of vanadium here can be considered a covariate that is obtained for each case with measurement error. Extending the nonlinear model in [5], we propose the nonlinear beta regression model with measurement errors given by

$$\begin{array}{c} g_{1}\left(\mu_{t}\right)=\alpha_{1}+\alpha_{2}\frac{z_{1t}}{z_{1t}+\alpha_{3}}+\alpha_{4}z_{2t}+\beta_{1}x_{1t},\;\log(\phi )=\gamma_{1},\;t=1,\ldots ,28, \end{array}$$ (13)

where *x*_1*t*_ represents vanadium concentration, *z*_1*t*_ denotes water steam and *z*_2*t*_ is a categorical variable indicating in which of the two temperatures the experiment was done (0 = 700 °*C* and 1 = 760 °*C*). Since *x*_1*t*_ is a covariate with measurement error, we need to previously estimate the nuisance parameters $\mu_{x},\sigma_{x}^{2}$ and $\sigma_{e}^{2}$. Thus, we perform a simple linear regression for the relation between the original *x* and observed *w* chemical complexes, as *w*_*t*_ = *τ*_1_ + *τ*_2_ *x*_*t*_ + *e*_*t*_, with *t* = 1, …, 15, $e_{t}∼N\left(0,\sigma_{e}^{2}\right)$. The obtained estimates were ${\hat{\tau }}_{1}=0.181$ (0.123), ${\hat{\tau }}_{2}=0.713$ (0.089) and ${\hat{\sigma }}_{e}^{2}=0.03810$, with *R*^2^ = 0.98. Also, we have ${\hat{\sigma }}_{x}^{2}=0.68$, supposing $x_{t}∼N\left(\mu_{x},\sigma_{x}^{2}\right)$. This yields an estimate of the reliability coefficient $k_{x}=\sigma_{x}^{2}/\left(\sigma_{x}^{2}+\sigma_{e}^{2}\right)$ of ${\hat{k}}_{x}=0.9436$, a low measurement error.

We have tried two different link functions: the logit and the complementary log-log. According to [17], when the mean of the response variable is near 1, the complementary log-log link function for the mean softens the impact of influent points in maximum likelihood estimation. In this sense, we consider here the logit and the complementary log-log link functions and check the performances of both modelings.

Tables (4) and (5) present the lower and upper limits of the estimated confidence intervals for the parameters of Model (13) obtained for the confidence levels of 90%, 95% and 99%, by considering the logit and complementary log-log link functions, respectively. We also present the lengths of the interval estimates. From both tables, it can be seen that the only interval estimate having zero is that for *α*_2_ at the nominal level of 99% using the method of regression calibration and with the logit model for the mean. Note that by accepting the hypothesis that *α*_2_ = 0, we eliminate the term $\alpha_{2}\frac{z_{1t}}{z_{1t}+\alpha_{3}}$ from the model and, consequently, not only the covariate *z*_1*t*_, but also nonlinearity. Also, observe that the length 0.245 of the confidence interval is quite large, more than twice the length of 0.0936 for the confidence interval of this same parameter and method of estimation, but using the complementary log-log as the link function.

**Table 4:** Lower Limits (LL), upper limits (UL) and length (length) of interval estimations obtained considering 90%, 95% and 99% for confidence level, using as estimation methods *ι*_*Naive*_, regression calibration (*ι*_*rc*_), approximate maximum likelihood (*ι*_*a*_) and approximate pseudo maximum likelihood (*ι*_*p*_). Logit link function. Fluid Catalytic Cracking (FCC) application.

Methods	Parameters	CI 90%			CI 95%			CI 99%		
		LL	UL	length	LL	UL	length	LL	UL	length
*ι*_*Naive*_	*α*_1_	2.05259	2.47829	0.42570	2.01182	2.51907	0.50725	1.93212	2.59876	0.66664
	*α*_2_	−0.15852	−0.04847	0.11005	−0.16906	−0.03793	0.13113	−0.18966	−0.01733	0.17234
	*α*_3_	−30.85159	−21.42127	9.43032	−31.75489	−20.51797	11.23692	−33.52034	−18.75252	14.76782
	*α*_4_	−0.53894	−0.16029	0.37865	−0.57521	−0.12402	0.45119	−0.64610	−0.05314	0.59296
	*β*_1_	−0.72235	−0.39372	0.32863	−0.75383	−0.36225	0.39159	−0.81536	−0.30072	0.51463
	*γ*_1_	3.82316	4.69967	0.87650	3.73921	4.78362	1.04442	3.57512	4.94771	1.37260
*ι*_*rc*_	*α*_1_	2.07344	2.50673	0.43329	2.03194	2.54823	0.51630	1.95082	2.62935	0.67853
	*α*_2_	−0.18173	−0.02527	0.15646	−0.19671	−0.01028	0.18643	−0.22600	0.01901	0.24502
	*α*_3_	−30.34481	−21.92805	8.41676	−31.15103	−21.12183	10.02919	−32.72673	−19.54613	13.18059
	*α*_4_	−0.61799	−0.08124	0.53675	−0.66940	−0.02983	0.63957	−0.76989	0.07065	0.84054
	*β*_1_	−0.74831	−0.44067	0.30764	−0.77778	−0.41120	0.36658	−0.83537	−0.35361	0.48176
	*γ*_1_	3.69926	4.82357	1.12431	3.59157	4.93126	1.33969	3.38109	5.14174	1.76066
*ι*_*a*_	*α*_1_	2.07119	2.51090	0.43971	2.02907	2.55302	0.52395	1.94675	2.63534	0.68859
	*α*_2_	−0.15887	−0.04902	0.10985	−0.16939	−0.03850	0.13089	−0.18996	−0.01793	0.17202
	*α*_3_	−30.83006	−21.37182	9.45824	−31.73603	−20.46584	11.27019	−33.50671	−18.69517	14.81154
	*α*_4_	−0.54651	−0.16333	0.38319	−0.58322	−0.12662	0.45660	−0.65496	−0.05489	0.60007
	*β*_1_	−0.76658	−0.41297	0.35361	−0.80045	−0.37910	0.42135	−0.86665	−0.31290	0.55375
	*γ*_1_	3.85072	4.79551	0.94480	3.76022	4.88601	1.12579	3.58334	5.06288	1.47954
*ι*_*p*_	*α*_1_	2.04896	2.53316	0.48420	2.00258	2.57954	0.57696	1.91193	2.67018	0.75825
	*α*_2_	−0.14984	−0.05805	0.09179	−0.15863	−0.04926	0.10938	−0.17582	−0.03207	0.14375
	*α*_3_	−29.30830	−22.89357	6.41473	−29.92275	−22.27912	7.64362	−31.12365	−21.07822	10.04542
	*α*_4_	−0.53200	−0.17784	0.35415	−0.56592	−0.14392	0.42200	−0.63222	−0.07762	0.55460
	*β*_1_	−0.76217	−0.41743	0.34474	−0.79519	−0.38441	0.41078	−0.85973	−0.31987	0.53986
	*γ*_1_	3.86546	4.78077	0.91531	3.77779	4.86844	1.09066	3.60643	5.03980	1.43337

**Table 5:** Lower Limits (LL), upper limits (UL) and length (length) of interval estimations obtained considering 90%, 95% and 99% for confidence level, using as estimation methods *ι*_*Naive*_, regression calibration (*ι*_*rc*_), approximate maximum likelihood (*ι*_*a*_) and approximate pseudo maximum likelihood (*ι*_*p*_). Complementary log-log link function. Fluid Catalytic Cracking (FCC) application.

Methods	Parameters	CI 90%			CI 95%			CI 99%		
		LL	UL	length	LL	UL	length	LL	UL	length
*ι*_*Naive*_	*α*_1_	0.80588	0.98914	0.18326	0.78833	1.00670	0.21837	0.75402	1.04101	0.28698
	*α*_2_	−0.07247	−0.02467	0.04781	−0.07705	−0.02009	0.05696	−0.08600	−0.01114	0.07486
	*α*_3_	−30.96676	−22.02752	8.93923	−31.82302	−21.17126	10.65176	−33.49653	−19.49775	13.99878
	*α*_3_	−0.27015	−0.09198	0.17818	−0.28722	−0.07491	0.21231	−0.32058	−0.04155	0.27902
	*β*_1_	−0.35046	−0.19772	0.15274	−0.36509	−0.18309	0.18200	−0.39368	−0.15450	0.23919
	*γ*_1_	3.88034	4.75715	0.87681	3.79636	4.84114	1.04478	3.63221	5.00528	1.37307
*ι*_*rc*_	*α*_1_	0.81184	1.00739	0.19555	0.79311	1.02612	0.23301	0.75651	1.06273	0.30623
	*α*_2_	−0.07846	−0.01868	0.05979	−0.08419	−0.01295	0.07124	−0.09539	−0.00176	0.09363
	*α*_3_	−30.67078	−22.32350	8.34728	−31.47034	−21.52395	9.94640	−33.03303	−19.96125	13.07178
	*α*_4_	−0.31937	−0.04276	0.27661	−0.34586	−0.01627	0.32960	−0.39765	0.03552	0.43316
	*β*_1_	−0.36819	−0.21580	0.15240	−0.38279	−0.20120	0.18159	−0.41132	−0.17267	0.23865
	*γ*_1_	3.78844	4.84905	1.06060	3.68685	4.95064	1.26379	3.48830	5.14919	1.66090
*ι*_*a*_	*α*_1_	0.81567	1.00411	0.18844	0.79762	1.02216	0.22454	0.76235	1.05744	0.29509
	*α*_2_	−0.07258	−0.02485	0.04773	−0.07715	−0.02028	0.05687	−0.08609	−0.01135	0.07474
	*α*_3_	−30.93200	−21.97158	8.96042	−31.79029	−21.11329	10.67700	−33.46777	−19.43581	14.03196
	*α*_4_	−0.27103	−0.09222	0.17881	−0.28815	−0.07509	0.21306	−0.32163	−0.04162	0.28001
	*β*_1_	−0.37361	−0.20958	0.16403	−0.38932	−0.19387	0.19546	−0.42003	−0.16316	0.25687
	*γ*_1_	3.92316	4.88551	0.96235	3.83098	4.97769	1.14671	3.65082	5.15786	1.50703
*ι*_*p*_	*α*_1_	0.80695	1.01283	0.20588	0.78723	1.03256	0.24533	0.74869	1.07110	0.32241
	*α*_2_	−0.06756	−0.02987	0.03769	−0.07117	−0.02626	0.04492	−0.07823	−0.01920	0.05903
	*α*_3_	−29.56210	−23.34146	6.22064	−30.15796	−22.74561	7.41235	−31.32252	−21.58104	9.74148
	*α*_4_	−0.26970	−0.09354	0.17616	−0.28658	−0.07667	0.20991	−0.31956	−0.04369	0.27587
	*β*_1_	−0.37307	−0.21012	0.16296	−0.38868	−0.19451	0.19417	−0.41919	−0.16400	0.25519
	*γ*_1_	3.93373	4.87495	0.94122	3.84357	4.96511	1.12153	3.66737	5.14131	1.47395

In fact, it is remarkable how the complementary log-log model presents better interval estimation with confidence intervals having lengths much smaller than the corresponding ones for the logit model, in all cases. For example, for *α*_3_, the coefficient related to temperature, when the nominal confidence level is 95% and we use approximate pseudo maximum likelihood, the lengths of the interval estimates are 0.4220 and 0.2099 for the logit and complementary log-log models, respectively. Comparing the estimation methods, it is also remarkable that approximate pseudo maximum likelihood yields interval estimates with lengths, that are, in general, smaller than those of the interval estimates obtained with the other estimation methods. For example, using the complementary log-log link function, the confidence intervals for *α*_3_, the parameter that makes the model nonlinear, the associated lengths for *ι*_*p*_ are (6.22, 7.41, 9.74), while those associated to *ι*_*a*_, *ι*_*rc*_ and *ι*_*naive*_ are, respectively, (8.96, 10.68, 14.03); (8.35, 9.95, 13.07); (8.94, 10.65, 13.99).

It is important here to observe that approximate pseudo maximum likelihood presents much better performance than the naive model, that which does not take measurement error into account, even for a very low measurement error, that is ${\hat{k}}_{x}\approx 0.95$. This shows how important to consider measurement errors can be when estimating the beta nonlinear regression models.

From all the results here presented, we select for this particular data set the nonlinear beta regression model for which the covariate *x*_1*t*_, the vanadium concentration, has measurement error, and the regression structure defined by

$$\log[-\log(1-\mu_{t})]=\alpha_{1}+\alpha_{2}\frac{z_{1t}}{z_{1t}+\alpha_{3}}+\alpha_{4}z_{2t}+\beta_{1}x_{1t}\;\log(\phi )=\gamma_{1},\;t=1,\ldots ,28.$$

Inference for measurement error in *x*_1*t*_ produces ${\hat{\sigma }}_{e}^{2}=0.03810$, ${\hat{\sigma }}_{x}^{2}=0.68$, ${\hat{k}}_{x}\approx 0.95$ and estimation based on approximate pseudo maximum likelihood yields ${\hat{\alpha }}_{1}$, ${\hat{\alpha }}_{2}$, ${\hat{\alpha }}_{3}$, ${\hat{\beta }}_{1}$, ${\hat{\gamma }}_{1}$ equal to 0.910, −0.049, −26.451, −0.182, −0.292 and 4.405, respectively, with standard errors given by 0.063, 0.011, 1.891, 0.054, 0.050 and 0.286, all parameters being significant at the 1% level. Finally, note that $\hat{\phi }=\exp\left({\hat{\gamma }}_{1}\right)\approx 82$ which for the beta regression can be considered a moderate precision, neither too low, but far from being considered high. That is, the application shows similarities with Scenario 1 of the simulations, for *ϕ* = 55 and *K*_*x*_ = 0.95. The results of the application only confirm those of the simulations in which, among the methods evaluated, the approximate pseudo maximum likelihood method presents the best performance regarding interval estimation.

## 5 Conclusions

We have proposed in this work a beta regression model with parameter nonlinearity and covariates measurement with errors for mean and precision submodels. Log-likelihood for this kind of model are written in terms of integrals with difficult solutions. For this reason, we have proposed to approximate those integrals using a Gaussian quadrature. This yields the estimators which we have referred to in this paper as the approximate maximum likelihood estimators. Because the approximate likelihood can become a very difficult function to maximize, we have also considered an approximate pseudo maximum likelihood, where we first estimate the nuisance parameters. The advantage of this two-phase method is to reduce the dimension of the problem, which makes maximization problem easier. We have also tried a regression calibration method, where the nonobserved variable is replaced by its conditional expectation in the likelihood function.

Numerical simulations have shown that the approximate pseudo maximum likelihood proposal is a very good alternative to estimate the model parameters, competing with the original maximum likelihood estimators. On the other hand, regression calibration is not a good proposal, which means that too much information about the observations can be truncated when we use this method. Therefore, regression calibration is not recommended here, although we strongly recommend approximate pseudo maximum likelihood.

An application has also shown that approximate pseudo maximum likelihood presents much better performance than the naive model, which does not take measurement error into account, even for very low measurement error. This shows how important to consider measurement errors can be when estimating beta nonlinear regression models. Furthermore, noteworthy are the superior performances related to the use of complementary log-log link function, in a sample whose responses are close to the upper limit of the unit interval.

## 6 Appendix: Approximate maximum likelihood

Differentiating the approximate log-likelihood *ℓ*_*a*_(**Ψ**) defined em (10) with respect to each one of the interest parameters *θ* = (*α*^⊤^, *β*, *γ*^⊤^, λ)^⊤^, we obtain the approximate score function, given by $U_{a}\left(\theta^{⊤}\right)={\left(U_{\alpha }^{⊤},U_{\beta },U_{\gamma }^{⊤},U_{\lambda }\right)}^{⊤}$ with $U_{\alpha }(\theta )=F_{1}^{⊤}BD_{1}$, $U_{\beta }\left(\theta \right)={D_{2}}^{⊤}b$, $U_{\gamma }\left(\theta \right)=F_{3}^{⊤}BD_{3}$ and $U_{\lambda }\left(\theta \right)={D_{4}}^{⊤}b$ Also, *B* is a *n* × *n* diagonal matrix in which the *t*-th diagonal element is given by

$$\begin{array}{c} b_{t}={\left(\sum_{q=1}^{Q}\frac{\nu_{q}}{\sqrt{\pi }}\exp\left\{\ell_{tq}\left(\mu_{tq},\phi_{tq}\right)\right\}\right)}^{-1},\;D_{1}=\left[\left(E\circ P\right)\circ H_{1}\right]V_{q},\;D_{3}=\left[\left(E\circ A\right)\circ H_{2}\right]V_{q}, \end{array}$$ (14)

with **E**, **A**, **H**_1_, **H**_2_, and **P** being *n* × *Q* matrices with entries defined as *e*_*tq*_ = exp{*ℓ*_*tq*_(*μ*_*tq*_, *ϕ*_*tq*_)},

$$\begin{array}{ccc} a_{tq} & = & \mu_{tq}\left(y_{t}^{*}-\mu_{tq}^{*}\right)+\log\left(1-y_{t}\right)-\psi \left(\left(1-\mu_{tq}\right)\phi_{tq}\right)+\psi \left(\phi_{tq}\right)\;\text{with} \end{array}$$ (15)

$$\begin{array}{ccc} y_{t}^{*} & = & \log(\frac{y_{t}}{1-y_{t}}),\;\mu_{tq}^{*}=\psi \left(\mu_{tq}\phi_{tq}\right)-\psi \left[\left(1-\mu_{tq}\right)\phi_{tq}\right], \end{array}$$ (16)

$h_{1tq}=1/g_{1}^{\prime }\left(\mu_{tq}\right)$, $h_{2tq}=1/g_{2}^{\prime }\left(\phi_{tq}\right)$ and $p_{tq}=\phi_{tq}\left(y_{t}^{*}-\mu_{tq}^{*}\right)$, respectively, where *ψ*(⋅) represents the digamma function (first derivative of log of gamma function). Additionally, **V**_*q*_ is a *Q*-dimensional vector with *q*-th element given by $\nu_{q}/\sqrt{\pi }$ where *ν*_*t*_ is the weight for the orthogonal Hermite polynomial of order *Q* in (9). Finally, we have *D*_2_ = [(*E*∘*P*)∘(*H*_1_∘*F*_2_)]*V*_*q*_ and *D*_4_ = [(*E*∘*A*)∘(*H*_2_∘*F*_4_)]*V*_*q*_.

The approximate score vector $U_{a}\left(\xi^{⊤}\right)={\left(U_{\mu_{x}},U_{\sigma_{x}^{2}}\right)}^{⊤}$ for the nuisance parameters is given by

$$\begin{array}{c} U_{\mu_{x}}=\frac{1}{\left(\sigma_{x}^{2}+\sigma_{e}^{2}\right)}\sum_{t=1}^{n}\left(w_{t}-\mu_{x}\right)+\sum_{t=1}^{n}b_{t}a_{t\mu_{x}},U_{\sigma_{x}^{2}}=-\frac{1}{2(\sigma_{x}^{2}+\sigma_{e}^{2})}\sum_{t=1}^{n}[1-\frac{{\left(w_{t}-\mu_{x}\right)}^{2}}{\left(\sigma_{x}^{2}+\sigma_{e}^{2}\right)}]+\sum_{t=1}^{n}b_{t}a_{t\sigma_{x}^{2}},\\ a_{t\mu_{x}}=\sum_{q=1}^{Q}\frac{\nu_{q}}{\sqrt{\pi }}\exp\left\{\ell_{tq}\left(\mu_{tq},\phi_{tq}\right)\right\}[\frac{\phi_{tq}\left(y_{t}^{*}-\mu_{tq}^{*}\right)}{g_{1}^{\prime }\left(\mu_{tq}\right)}\frac{\partial \eta_{1t}}{\partial \mu_{x}}+\frac{a_{tq}}{g_{2}^{\prime }\left(\phi_{tq}\right)}\frac{\partial \eta_{2t}}{\partial \mu_{x}}];\\ a_{t\sigma_{x}^{2}}=\sum_{q=1}^{Q}\frac{\nu_{q}}{\sqrt{\pi }}\exp\left\{\ell_{tq}\left(\mu_{tq},\phi_{tq}\right)\right\}[\frac{\phi_{tq}\left(y_{t}^{*}-\mu_{tq}^{*}\right)}{g_{1}^{\prime }\left(\mu_{tq}\right)}\frac{\partial \eta_{1t}}{\partial \sigma_{x}^{2}}+\frac{a_{tq}}{g_{2}^{\prime }\left(\phi_{tq}\right)}\frac{\partial \eta_{2t}}{\partial \sigma_{x}^{2}}]. \end{array}$$

We have that the approximate information matrix for the interest parameters is given by −*J*_*a*_(*θ*), with

$$\begin{array}{c} J_{a}\left(\theta \right)={\left[\begin{array}{cccc} \frac{\partial^{2}\ell_{a}\left(\Psi \right)}{\partial \alpha \partial \alpha^{⊤}} & \frac{\partial^{2}\ell_{a}\left(\Psi \right)}{\partial \alpha d\beta } & \frac{\partial^{2}\ell_{a}\left(\Psi \right)}{\partial \alpha \partial \gamma^{⊤}} & \frac{\partial^{2}\ell_{a}\left(\Psi \right)}{\partial \alpha d\lambda }\\ & \frac{\partial^{2}\ell_{a}\left(\Psi \right)}{d\beta d\beta } & \frac{\partial^{2}\ell_{a}\left(\Psi \right)}{d\beta \partial \gamma^{⊤}} & \frac{\partial^{2}\ell_{a}\left(\Psi \right)}{d\beta d\lambda }\\ & & \frac{\partial^{2}\ell_{a}\left(\Psi \right)}{\partial \gamma \partial \gamma^{⊤}} & \frac{\partial^{2}\ell_{a}\left(\Psi \right)}{\partial \gamma d\lambda }\\ & & & \frac{\partial^{2}\ell_{a}\left(\Psi \right)}{d\lambda d\lambda } \end{array}\right]}_{n(\theta )\times n(\theta )}, \end{array}$$

in which $n\left(\theta \right)=\left(p+1\right)+\left(\overset{ˇ}{q}+1\right)<n$ and for *i*, *j* = 1, 2, …, *p* (first row), *i* = 1, 2, …, *p*; $j=1,2,\ldots ,\overset{ˇ}{q}$ (second row), $j=1,2,\ldots ,\overset{ˇ}{q}$ (third row), $k=1,2,\ldots ,\overset{ˇ}{q}$ (the last rows) we have that

$$\begin{array}{cc} & \frac{\partial^{2}\ell_{a}\left(\Psi \right)}{\partial \alpha_{i}\partial \alpha_{j}}=J_{a(\alpha_{i}\alpha_{j})}=\sum_{t=1}^{n}[{\dot{b}}_{t(\alpha_{j})}a_{t\alpha_{i}}+b_{t}{\dot{a}}_{t\alpha_{i}\left(\alpha_{j}\right)}],\;\frac{\partial^{2}\ell_{a}\left(\Psi \right)}{\partial \alpha_{i}\partial \beta }=J_{a(\alpha_{i}\beta )}=\sum_{t=1}^{n}[{\dot{b}}_{t(\beta )}a_{t\alpha_{i}}+b_{t}{\dot{a}}_{t\alpha_{i}\left(\beta \right)}],\\ & \frac{\partial^{2}\ell_{a}\left(\Psi \right)}{\partial \alpha_{i}d\lambda }=J_{a(\alpha_{i}\lambda )}=\sum_{t=1}^{n}[{\dot{b}}_{t(\lambda )}a_{t\alpha_{i}}+b_{t}{\dot{a}}_{t\alpha_{i}\left(\lambda \right)}],\;\frac{\partial^{2}\ell_{a}\left(\Psi \right)}{\partial \alpha_{i}\partial \gamma_{j}}=J_{a(\alpha_{i}\gamma_{j})}=\sum_{t=1}^{n}[{\dot{b}}_{t(\gamma_{j})}a_{t\alpha_{i}}+b_{t}{\dot{a}}_{t\alpha_{i}\left(\gamma_{j}\right)}]\\ & \frac{\partial^{2}\ell_{a}\left(\Psi \right)}{d\beta d\beta }=J_{a(\beta \beta )}=\sum_{t=1}^{n}[{\dot{b}}_{t(\beta )}a_{t\beta }+b_{t}{\dot{a}}_{t\beta (\beta )}],\;\frac{\partial^{2}\ell_{a}\left(\Psi \right)}{d\beta \partial \gamma_{j}}=J_{a(\beta \gamma_{j})}=\sum_{t=1}^{n}[{\dot{b}}_{t(\gamma_{j})}a_{t\beta }+b_{t}{\dot{a}}_{t\beta (\gamma_{j})}],\\ & \frac{\partial^{2}\ell_{a}\left(\Psi \right)}{d\beta d\lambda }=J_{a(\beta \lambda )}=\sum_{t=1}^{n}[{\dot{b}}_{t(\lambda )}a_{t\beta }+b_{t}{\dot{a}}_{t\beta (\lambda )}],\;\frac{\partial^{2}\ell_{a}\left(\Psi \right)}{\partial \gamma_{k}\partial \gamma_{j}}=J_{a(\gamma_{k}\gamma_{j})}=\sum_{t=1}^{n}[{\dot{b}}_{t(\gamma_{j})}a_{t\gamma_{k}}+b_{t}{\dot{a}}_{t\gamma_{k}\left(\gamma_{j}\right)}]\\ & \frac{\partial^{2}\ell_{a}\left(\Psi \right)}{\partial \gamma_{k}d\lambda }=J_{a(\gamma_{k}\lambda )}=\sum_{t=1}^{n}[{\dot{b}}_{t(\lambda )}a_{t\gamma_{k}}+b_{t}{\dot{a}}_{t\gamma_{k}\left(\lambda \right)}],\;\frac{\partial^{2}\ell_{a}\left(\Psi \right)}{d\lambda d\lambda }=J_{a(\lambda \lambda )}=\sum_{t=1}^{n}[{\dot{b}}_{t(\lambda )}a_{t\lambda }+b_{t}{\dot{a}}_{t\lambda (\lambda )}]. \end{array}$$ (17)

Many expressions in (17) differ only in the parameter of interest. More specifically, they differ as to the derivatives of *η*_1_ and *η*_2_ with respect to these parameters. Thus, we defined a general argument *ϑ* varying among the interest parameters, namely: *α*_*i*_, *γ*_*j*_, *β*, λ. Furthermore, we also defined the following functions of *ϑ*:

$$\begin{array}{ccc} {\dot{\ell }}_{tq(\vartheta )}\left(\mu_{tq},\phi_{tq}\right) & = & \phi_{tq}\left[y_{t}^{*}-\mu_{tq}^{*}\right]\frac{1}{g_{1}^{\prime }\left(\mu_{tq}\right)}\frac{\partial \eta_{1t}}{\partial \vartheta },\;\text{for}\;\vartheta =\alpha_{i}\;\;\text{and}\;\;\beta ;\\ & = & a_{tq}\frac{1}{g_{2}^{\prime }\left(\phi_{tq}\right)}\frac{\partial \eta_{2t}}{\partial \vartheta },\;\text{for}\;\vartheta =\gamma_{j}\;\;\text{and}\;\;\lambda .\\ a_{t\vartheta } & = & \sum_{q=1}^{Q}\frac{\nu_{q}}{\sqrt{\pi }}\exp\left\{\ell_{tq}\left(\mu_{tq},\phi_{tq}\right)\right\}{\dot{\ell }}_{tq(\vartheta )}\left(\mu_{tq},\phi_{tq}\right),\;\text{for}\;\vartheta =\alpha_{i},\beta ,\gamma_{j},\lambda .\\ {\dot{b}}_{t(\vartheta )} & = & -b_{t}^{2}\sum_{q=1}^{Q}\frac{\nu_{q}}{\sqrt{\pi }}\exp\left\{\ell_{tq}\left(\mu_{tq},\phi_{tq}\right)\right\}{\dot{\ell }}_{tq(\vartheta )}\left(\mu_{tq}\phi_{tq}\right),\;\text{for}\;\vartheta =\alpha_{i},\beta ,\gamma_{j},\lambda .\\ {\dot{a}}_{t\alpha_{i}\left(\vartheta \right)} & = & \sum_{q=1}^{Q}\frac{\nu_{q}}{\sqrt{\pi }}\exp\left\{\ell_{tq}\left(\mu_{tq},\phi_{tq}\right)\right\}[{\dot{\ell }}_{tq(\alpha_{j})}\left(\mu_{tq},\phi_{tq}\right){\dot{\ell }}_{tq(\vartheta )}\left(\mu_{tq},\phi_{tq}\right)+{\ddot{\ell }}_{tq\left(\alpha_{i}\right)\left(\vartheta \right)}\left(\mu_{tq},\phi_{tq}\right)],\;\;\text{with}\\ {\ddot{\ell }}_{tq\left(\alpha_{i}\right)\left(\vartheta \right)}\left(\mu_{tq},\phi_{tq}\right) & = & -\phi_{tq}[u_{tq}+\left(y_{t}^{*}-\mu_{tq}^{*}\right)\frac{g_{1}^{\prime \prime }\left(\mu_{tq}\right)}{{\left[g_{1}^{\prime }\left(\mu_{tq}\right)\right]}^{3}}]\frac{\partial \eta_{1t}}{\partial \alpha_{i}}\frac{\partial \eta_{1t}}{\partial \vartheta };\;\text{for}\;\vartheta =\alpha_{j}\;\;\text{and}\;\;\beta ,\;\;i,j=1,\ldots ,p.\\ & = & [\left(y_{t}^{*}-\mu_{tq}^{*}\right)-c_{tq}]\frac{1}{g_{1}^{\prime }\left(\mu_{tq}\right)}\frac{1}{g_{2}^{\prime }\left(\phi_{tq}\right)}\frac{\partial \eta_{1t}}{\partial \alpha_{i}}\frac{\partial \eta_{2t}}{\partial \vartheta };\;\text{for}\;\vartheta =\gamma_{j}\;\;\text{and}\;\;\lambda ,\;\;j=1,\ldots ,\overset{ˇ}{q}.\\ {\dot{a}}_{t\beta (\vartheta )} & = & \sum_{q=1}^{Q}\frac{\nu_{q}}{\sqrt{\pi }}\exp\left\{\ell_{tq}\left(\mu_{tq},\phi_{tq}\right)\right\}[{\dot{\ell }}_{tq(\vartheta )}\left(\mu_{tq},\phi_{tq}\right){\dot{\ell }}_{tq(\beta )}\left(\mu_{tq},\phi_{tq}\right)+{\ddot{\ell }}_{tq(\beta )(\vartheta )}\left(\mu_{tq},\phi_{tq}\right)],\;\;\text{with}\\ {\ddot{\ell }}_{tq(\beta )(\vartheta )}\left(\mu_{tq},\phi_{tq}\right) & = & -\phi_{tq}[u_{tq}+\left(y_{t}^{*}-\mu_{tq}^{*}\right)\frac{g_{1}^{\prime \prime }\left(\mu_{tq}\right)}{{\left[g_{1}^{\prime }\left(\mu_{tq}\right)\right]}^{3}}]\frac{\partial \eta_{1t}}{\partial \beta }\frac{\partial \eta_{1t}}{\partial \beta };\;\text{for}\;\vartheta =\beta .\\ & = & [\left(y_{t}^{*}-\mu_{tq}^{*}\right)-c_{tq}]\frac{1}{g_{1}^{\prime }\left(\mu_{tq}\right)}\frac{1}{g_{2}^{\prime }\left(\phi_{tq}\right)}\frac{\partial \eta_{1t}}{\partial \beta }\frac{\partial \eta_{2t}}{\partial \vartheta };\;\text{for}\;\vartheta =\gamma_{j}\;\;\text{and}\;\;\lambda ,\;\;j=1,\ldots ,\overset{ˇ}{q}.\\ {\dot{a}}_{t\gamma_{k}\left(\vartheta \right)} & = & \sum_{q=1}^{Q}\frac{\nu_{q}}{\sqrt{\pi }}\exp\left\{\ell_{tq}\left(\mu_{tq},\phi_{tq}\right)\right\}[{\dot{\ell }}_{tq(\vartheta )}\left(\mu_{tq},\phi_{tq}\right){\dot{\ell }}_{tq(\gamma_{k})}\left(\mu_{tq},\phi_{tq}\right)+{\ddot{\ell }}_{tq\left(\gamma_{k}\right)\left(\vartheta \right)}\left(\mu_{tq},\phi_{tq}\right)],\;\;\text{with}\\ {\ddot{\ell }}_{tq\left(\gamma_{k}\right)\left(\vartheta \right)}\left(\mu_{tq},\phi_{tq}\right) & = & \{d_{tq}-a_{tq}\frac{g_{2}^{\prime \prime }\left(\phi_{tq}\right)}{{\left[g_{2}^{\prime }\left(\phi_{tq}\right)\right]}^{3}}\}\frac{\partial \eta_{2t}}{\partial \gamma_{k}}\frac{\partial \eta_{2t}}{\partial \vartheta };\;\text{for}\;\vartheta =\lambda \;\;\text{and}\;\;\gamma_{j},\;\;k,j=1,\ldots ,\overset{ˇ}{q}.\\ {\dot{a}}_{t\lambda (\lambda )} & = & \sum_{q=1}^{Q}\frac{\nu_{q}}{\sqrt{\pi }}\exp\left\{\ell_{tq}\left(\mu_{tq},\phi_{tq}\right)\right\}[{\dot{\ell }}_{tq(\lambda )}\left(\mu_{tq},\phi_{tq}\right){\dot{\ell }}_{tq(\lambda )}\left(\mu_{tq},\phi_{tq}\right)+{\ddot{\ell }}_{tq(\lambda )(\lambda )}\left(\mu_{tq},\phi_{tq}\right)],\;\;\text{with}\\ {\ddot{\ell }}_{tq(\lambda )(\lambda )}\left(\mu_{tq},\phi_{tq}\right) & = & \{d_{tq}-a_{tq}\frac{g_{2}^{\prime \prime }\left(\phi_{tq}\right)}{{\left[g_{2}^{\prime }\left(\phi_{tq}\right)\right]}^{3}}\}\frac{\partial \eta_{2t}}{\partial \lambda }\frac{\partial \eta_{2t}}{\partial \lambda }. \end{array}$$ (18)

In (18) we have that *a*_*t*_ and *b*_*t*_ are defined in (14) and (15), respectively and $u_{tq}=\phi_{tq}\{\psi^{\prime }\left(\mu_{tq}\phi_{tq}\right)+\psi^{\prime }\left[\left(1-\mu_{tq}\right)\phi_{tq}\right]\}{\left[g_{1}^{\prime }\left(\mu_{tq}\right)\right]}^{-2}$, *c*_*tq*_ = *ϕ*_*tq*_[*ψ*′(*μ*_*tq*_ *ϕ*_*tq*_)*μ*_*tq*_ + *ψ*′[(1 − *μ*_*tq*_)*ϕ*_*tq*_](1 − *μ*_*tq*_)] and *d*_*tq*_ = *μ*_*tq*_{−*μ*_*tq*_ *ψ*′(*μ*_*tq*_ *ϕ*_*tq*_) − (1 − *μ*_*tq*_)*ψ*′[(1 − *μ*_*tq*_)*ϕ*_*tq*_]} + *ψ*′(*ϕ*_*tq*_) − (1 − *μ*_*tq*_)*ψ*′[(1 − *μ*_*tq*_)*ϕ*_*tq*_].

## Supporting information

S1 File We create a directory in to allocate the supplementary information, namely: “S1 File”. As supplementary material we placed two simulation programs, the application program, the data set, the file of quadrature points, and the description of the data set.
(ZIP)
