# Supplementary material for: Beta regression model nonlinear in the parameters with additive measurement errors in variables
Source: PLoS One. 2021 Jul 29;16(7):e0254103. doi: 10.1371/journal.pone.0254103 (PMC8320978; doi:10.1371/journal.pone.0254103)
Supplement: S1 File — As supplementary material we placed two simulation programs, the application program, the data set, the file of quadrature points, and the description of the data set. (ZIP) [file pone.0254103.s001.zip › SupportingInformation/Readme_data.pdf]

DATA DESCRIPTION

PATRÍCIA L. ESPINHEIRA

Description of data in the paper, x_{1t} represents vanadium concentration, z_{1t} denotes water steam and z_{2t} is a categorical variable indicating in which of the two temperatures the experiment was done (0 = 700 ^0C and 1 = 760 ^0C).

- First column is the Response Variable,
- Second column the is a categorical variable indicating temperatures.
- Third column water steam.
- Fourth column the temperature values (Not used in the application).
- Fifth column the vanadium concentration.
